# Expression Profiling of RNA Transcripts during Neuronal Maturation and Ischemic Injury

**DOI:** 10.1371/journal.pone.0103525

**Published:** 2014-07-25

**Authors:** Prameet Kaur, Dwi Setyowati Karolina, Sugunavathi Sepramaniam, Arunmozhiarasi Armugam, Kandiah Jeyaseelan

**Affiliations:** 1 Department of Biochemistry and Neuroscience Research Centre, Centre for Translational Medicine, Yong Loo Lin School of Medicine, National University of Singapore, Singapore, Singapore; 2 Department of Anatomy and Developmental Biology, School of Biomedical Sciences, Faculty of Medicine, Nursing and Health Sciences, Monash University, Clayton, Victoria, Australia; University of Missouri, United States of America

## Abstract

Neuronal development is a pro-survival process that involves neurite growth, synaptogenesis, synaptic and neuronal pruning. During development, these processes can be controlled by temporal gene expression that is orchestrated by both long non-coding RNAs and microRNAs. To examine the interplay between these different components of the transcriptome during neuronal differentiation, we carried out mRNA, long non-coding RNA and microRNA expression profiling on maturing primary neurons. Subsequent gene ontology analysis revealed regulation of axonogenesis and dendritogenesis processes by these differentially expressed mRNAs and non-coding RNAs. Temporally regulated mRNAs and their associated long non-coding RNAs were significantly over-represented in proliferation and differentiation associated signalling, cell adhesion and neurotrophin signalling pathways. Verification of expression of the *Axin2, Prkcb, Cntn1, Ncam1, Negr1, Nrxn1* and *Sh2b3* mRNAs and their respective long non-coding RNAs in an *in vitro* model of ischemic-reperfusion injury showed an inverse expression profile to the maturation process, thus suggesting their role(s) in maintaining neuronal structure and function. Furthermore, we propose that expression of the cell adhesion molecules, *Ncam1* and *Negr1* might be tightly regulated by both long non-coding RNAs and microRNAs.

## Introduction

Neuronal development is a tightly regulated multi-step process. Neural stem cells proliferate, differentiate and mature to give rise to the neuronal morphology and fully functional neurons [1]. Timely maturation of neurons, characterized by axonal and dendritic outgrowth, synaptogenesis, synaptic and neuronal pruning, modulation of neurotransmitter sensitivities and myelination, determines neuronal connections with extraordinary precision [2], [3]. These culminate into large, integrated networks of synapses with specific functions in the brain [4]–[6].

Gene expression throughout the neuronal maturation process is intricately regulated by distinct temporal and spatial expression of specific non-coding RNAs (ncRNAs), namely microRNAs (miRNAs) and long ncRNAs (lncRNAs) [7], [8]. miRNAs, the most well-characterized ncRNAs, are short endogenous molecules, approximately 22 nucleotides in length. In general, these small ncRNAs interact with their target mRNAs by complementary binding to bring about transcriptional and translational regulation [9], [10]. Brain-specific and brain-enriched miRNAs, miR-124 and miR-134, are vital regulators of neuronal functions associated with neurogenesis and synaptic plasticity respectively [11], [12]. LncRNAs, on the other hand, are transcripts longer than 200 nucleotides [13]. These RNA molecules coordinate gene expression through epigenetic modification, mRNA splicing, control of transcription or translation and genomic imprinting [14]. LncRNAs have been shown to play a role in embryogenesis and development of the central nervous system [7].

Several studies have demonstrated that ncRNAs that direct neuronal gene expression are dysregulated in neurovascular diseases such as stroke [15]–[17]. For instance, the expression of genes essential to axonal extension and neuronal survival, such as the cell adhesion molecule *NB-3*, is inversely regulated during ischemic injury, resulting in impaired neuronal survival and neurite outgrowth [18]. Moreover, modulation of certain miRNAs has been shown to confer neuroprotection in cerebral ischemic models [19]–[21]. Nevertheless, the exact role of lncRNAs in ischemic disease warrants further investigation. In this study we examine the roles of lncRNAs and miRNAs in controlling the expression of neuron specific mRNAs during neuronal maturation.

## Methods

### Primary cortical neuronal cultures

The experimental mice were handled according to National University of Singapore (IACUC/NUS) guidelines for laboratory animals. The protocol was approved by the Committee on the Ethics of Animal Experiments of the National University of Singapore (Protocol Number: 025/11).

**Figure 1:**
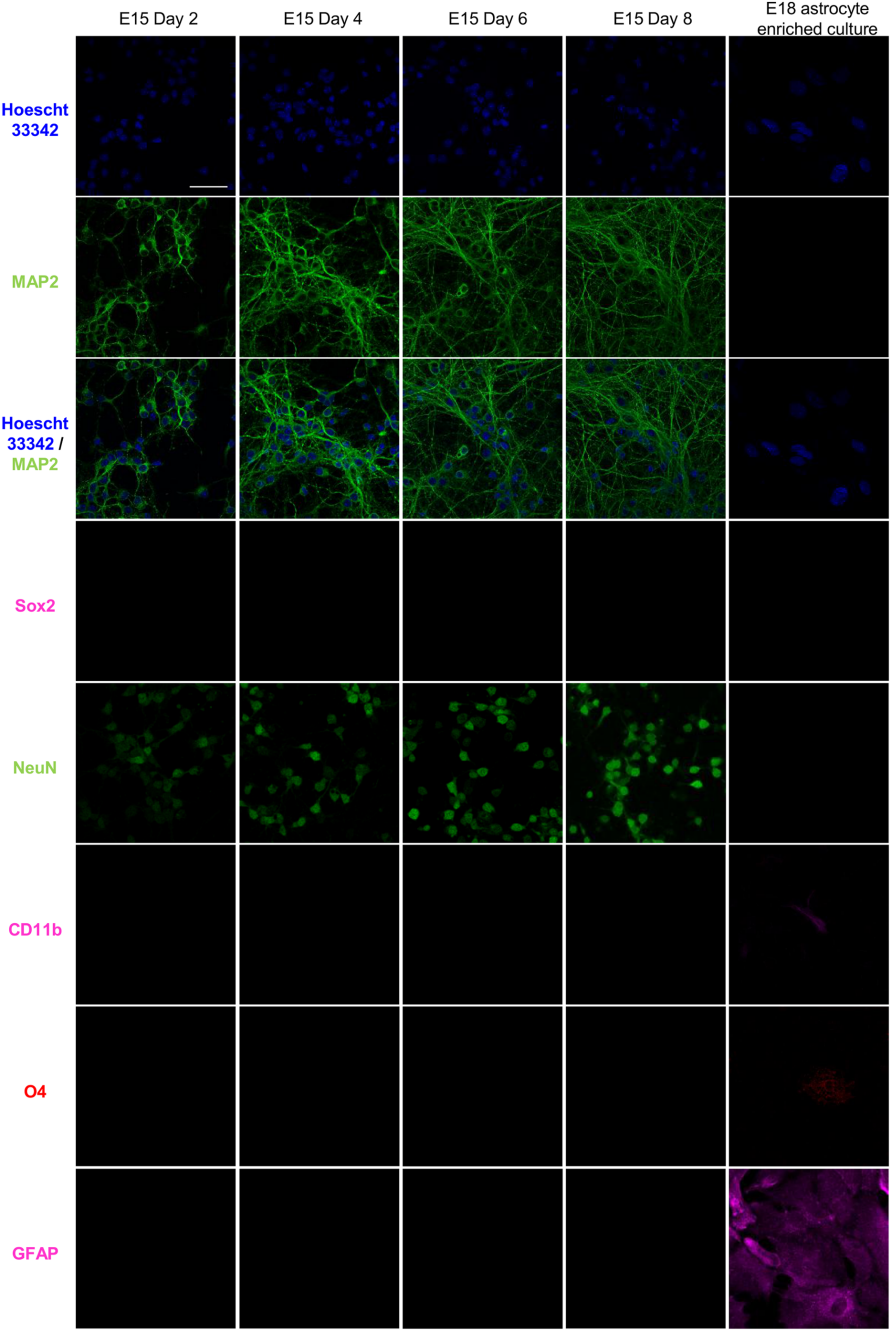
Maturation of E15 derived mouse primary cortical neuronal cultures. Cultures were immunostained for neuronal markers (MAP2 and NeuN), a neuronal progenitor marker (Sox2), microglial marker (CD11b), oligodendrocyte marker (O4) and astrocyte marker (GFAP). E15 cultures displayed absence of neuronal progenitor cells and glial cells, containing >99% neuronal cells. Short neurites at the early stages of development (Day 2) gave rise to an extensive network of neurites by day 8. E18 astrocyte enriched cultures were used as positive control for CD11b, O4 and GFAP staining. Scale bar represents 50 µm.

Murine cortical neurogenesis occurs mainly from embryonic day 10 (E10) to E18 and gliogenesis starts from E15 through early postnatal ages [22]–[24]. Hence, primary cultures of cortical neurons were established from E15 Swiss albino mouse brains according to the published protocols [25]–[28]. Cultures from E14 and E16 Swiss albino mouse brains were also included in this study as controls. Cortices were dissected from E15 mouse embryos and rinsed with Hanks’ balanced salt solution (HBSS, 14025-092, Gibco, Invitrogen, USA). The cortical slices were dissociated in HBSS containing 0.05% (w/v) trypsin without Ca^2+^/Mg^2+^ (14175-095, Gibco, Invitrogen, USA) for 30 min at 37°C [29], followed by neutralization in 1 mg/ml trypsin inhibitor (T6522, Sigma, USA) [26]. Single cells were obtained by gentle trituration in Neurobasal medium (21103-049, Gibco, Invitrogen, USA) supplemented with B27 (17504-044, Invitrogen, USA), L-glutamine and Penicillin-Streptomycin (Gibco, Invitrogen, USA) [25]. The cells were counted by trypan blue exclusion and seeded on to poly-d-lysine coated 24 well plates at a density of 120,000 cells/cm^2^ in Neurobasal medium supplemented with B27, L-glutamine and Penicillin-Streptomycin. Maintenance of primary neurons in Neurobasal medium supplemented with B27 is reported to reduce glia growth to less than 0.5% of the neuronal population ensuring a pure neuronal culture [25], [30]. Cultures were maintained at 37°C with 5% CO_2_ in a tissue culture incubator. Immunocytochemical staining of the cultures for neuronal markers (microtubule-associated protein 2, MAP2 and neuronal nuclei, NeuN) [31], a neuronal progenitor marker (SRY (sex determining region Y)-box 2, Sox2) [32], microglial marker (CD11b) [33], oligodendrocyte marker (O4) [34] and astrocyte marker (glial fibrillary acidic protein, GFAP) [35] were used to ensure the purity of neuronal cultures with no contamination from glial cells [26], [36]. Quantitation of miR-124, miR-143 and miR-223 were also carried out to confirm the neuronal nature of the primary culture [37]. Neuronal purity was determined by calculating the ratio of MAP2 positive cells to total viable cells. These data indicated >99% of the cells were neurons in our cultures (Figure 1, Table 1).

**Table 1:** Expression level of miR-124, miR-143 and miR-223, in primary cultures.

	Mean C_T_±SD		
	miR-124	miR-143	miR-223
**NTC**	39.0±0.3	34.4±0.9	38.4±0.6
**Neuronal culture**			
**E15 Day 2**	21.1±0.7**	32.3±1.6	36.3±0.2
**E15 Day 4**	21.0±0.7**	32.6±0.7	38.4±0.2
**E15 Day 6**	21.1±0.3**	33.9±1.0	39.0±0.4
**E15 Day 8**	21.0±0.0**	33.0±0.0	38.8±0.4
**0 hr OGD**	21.7±0.8**	34.1±0.3	39.3±0.2
**2 hr OGD**	21.3±0.1**	33.6±0.4	38.8±0.4
**4 hr OGD**	21.1±0.1**	34.1±0.6	39.3±0.3
**Astrocyte enriched culture**			
**E18**	38.5±0.6	30.4±0.0**	33.0±0.2**

qPCR was carried out on 10 ng RNA from maturing neurons and neurons subjected to OGD to determine the purity of the primary cortical neuronal cultures. qPCR amplification was carried out for a maximum of 40 cycles. miR-124 was found to be significantly expressed in neuronal cultures +/− OGD. miR-143 and miR-223 were found to be significantly expressed only in astrocyte enriched cultures. Expression is shown as mean C_T_±SD. Three technical replicates and 3 biological replicates were used as described in the methods section. Statistical significance were evaluated using the Student’s *t-test* (**p<0.01).

### Oxygen-glucose deprivation

Primary neuronal cultures from day 6 were subjected to oxygen-glucose deprivation (OGD) conditions as previously described [38]. Glucose-free Earle’s balanced salt solution (EBSS) was saturated with a mixture of 5% CO_2_, 95% N_2_, in a ProOx *in vitro* chamber (BioSpherix, USA) with O_2_ maintained at 0.1%, at 37°C overnight. Day 6 neuronal cultures were washed twice with this medium and incubated for 2, 4, 6, 8 hr in the hypoxic chamber. OGD was terminated by replacing the glucose-free EBSS with reperfusion medium (Neurobasal medium with L-glutamine and Penicillin-streptomycin, without B27 supplement). Control cultures were treated identically, but without exposure to OGD conditions. During reperfusion, the cells were maintained in a regular 5% CO_2_ incubator for 24 hr.

### Morphologic assessment of cell death

Cells subjected to OGD were stained with Hoechst 33342 and Ethidium Homodimer III (EtHD) dye (Biotium, USA.) according to the manufacturer’s protocol. Stained cells were protected from light until visualized by fluorescence microscopy (Leica DMIRB, Germany). Images were captured at 40× objectives and cell morphology was determined. A minimum of 3 fields of at least 100 cells per field was counted to determine the percentage of healthy cells from the total number of cells. Experiments were carried out in triplicates and repeated four different times (n = 4).

### Immunocytochemistry

For immunofluorescence studies, the cells were grown on sterile coverslips in 24-well plates. On specified days, the cells were fixed in 4% paraformaldehyde in phosphate-buffered saline for 20 minutes, permeabilized with 0.1% Triton X-100 for 30 min and then blocked with 5% FBS in PBS for 30 min at room temperature. The fixed cells were incubated with mouse anti-MAP2 (1∶500; Abcam, Cambridge, UK), mouse anti-NeuN (1∶1000; Abcam, Cambridge, UK), rabbit anti-Sox2 (1∶250; Abcam, Cambridge, UK), rabbit anti-CD11b (1∶500; Abcam, Cambridge, UK), rabbit anti-GFAP (1∶500; Abcam, Cambridge, UK) and mouse IgM anti-O4 (1∶100; Sigma, USA) primary antibodies for 1 hr. The cells were then incubated with FITC-, Texas Red- and Cy5-coupled secondary antibodies (1∶200; Abcam, Cambridge, UK) for 1 hr. Hoechst 33342 was used as a nuclear stain. Images were viewed and analyzed using LSM710 confocal imaging software (Carl Zeiss MicroImaging Inc, Germany).

### Extraction of total RNA

Total RNA (+ miRNA) was extracted from cells by a single-step method using Trizol Reagent (Invitrogen, Life Technologies, USA) according to the manufacturers’ protocol. An additional step was included to remove any residual DNA by treatment of the aqueous phase with 3 units of RNase free DNase I for 20 mins at 37°C. The purity of the RNA was determined using Nanodrop ND-2000c spectrophotometry (Nanodrop Tech, Rockland, Del) to ensure the 260/280 ratio was well within the 1.8–2.0 range. The concentration of the RNA was also determined using Nanodrop ND-2000c spectrophotometry and RNA integrity verified with denaturing agarose gel and denaturing polyacrylamide gel electrophoresis.

### Reverse Transcription and Quantitative PCR

Reverse transcription followed by real-time quantitative PCR (qPCR) were carried out according to Jeyaseelan et al. (2008) [15]. Reverse transcription was carried out using the Taqman RT reagents kit (Applied Biosystems, USA) on an ABI PRISM 7900 cycler according to the manufacturer’s protocol. Quantitation of mRNAs and their respective lncRNAs was performed using SYBR Green Assay (Applied Biosystems, USA). Specific primer sequences (Table S1) were generated using PrimerExpress software (Applied Biosystems, USA) and a PCR product dissociation curve was generated to ensure specificity of amplification. For miRNA detection, 10 ng total RNA was reverse transcribed using miRNA specific primers and stem-loop real-time qPCR performed according to the manufacturer’s protocol using Taqman miRNA assays for miR-124 (assay ID: 001182), miR-143 (assay ID: 002249) and miR-223 (assay ID: 002295) (Applied Biosystems, USA). qPCR amplification cycle was maintained at 40 and miRNAs with C_T_ (cycle threshold) values >35 were considered absent. For expression analysis, the mRNA, lncRNA and miRNA qPCR data was normalized to the endogenous control, GAPDH, followed by normalization to Day 2 cultures or 0 hr OGD control cultures using relative quantification (2∧-delta(delta Ct)). Results were generated from 3 technical replicates and 3 biological replicates for each mRNA/lncRNA/miRNA dataset. The average fold change ± standard deviation (SD) was determined and Student’s *t-test* was carried out to determine statistical significance.

For determination of purity of the neuronal cultures, the abundance of miR-124, -143 and -223 was determined based on only the C_T_ values as normalization to the endogenous control would yield fold change values which would not be a good reflection of absence or presence of the miRNA. qPCR can accurately quantify down to a single copy of an RNA/miRNA. Hence, C_T_ values were determined and Student’s t-test performed to determine if the obtained C_T_ values were significantly different from no template controls (NTC) which would indicate presence of the specific miRNA. All reactions were carried out on Applied Biosystems 7500 sequence detection system and analyzed by the Applied Biosystems 7500 real-time PCR system software version 2.0.6 according to the manufacturer’s protocol.

GAPDH is a commonly used housekeeping gene for qPCR normalization in neuronal differentiation [39], [40] as well as primary neuronal cultures subjected to OGD [41], [42]. In addition to this, to determine the most stable housekeeping gene for qPCR normalization, qPCR measurements for the GAPDH and β-actin were carried out in maturing neuronal cultures and neuronal cultures subjected to OGD. Student’s *t-test* showed that the C_T_ values for β-actin were significantly different between 2 hr OGD and 4 hr OGD samples. The expression of GAPDH, on the other hand, remained non-significant across all the time points, making it a more stable gene for normalization. Hence, we used GAPDH as an endogenous control for normalization of our qPCR data as it is stable in neurons subjected to OGD as well as in neuronal differentiation. Reverse transcription-qPCR assays were performed according to the MIQE guidelines [43], and respective information is provided in the supporting material (Table S2).

### LncRNA and mRNA arrays

The microarray profiling of the cortical neurons were carried out on Arraystar platform (Arraystar Inc., Rockville, USA). Sample labelling and array hybridization were performed according to the Agilent One-Color Microarray-Based Gene Expression Analysis protocol (Agilent Technologies, USA) by Arraystar, USA with minor modifications. Total RNA from 4 separate experiments (n = 4) was pooled for each time point (Day 2, Day 4, Day 6, Day 8, 0 hr OGD, 2 hr OGD, 4 hr OGD). Briefly, mRNA was purified from total RNA after removal of rRNA (mRNA-ONLY Eukaryotic mRNA Isolation Kit, Epicentre, USA). Then, each sample was amplified and transcribed into fluorescent cRNA along the entire length of the transcripts without 3′ bias utilizing a random priming method. The labelled cRNAs were purified by RNeasy Mini Kit (Qiagen, USA). The concentration and specific activity of the labelled cRNAs (pmol Cy3/µg cRNA) were measured by NanoDrop ND-1000. One µg of each labelled cRNA was fragmented by adding 5 µl 10× Blocking Agent (Agilent Technologies, USA) and 1 µl of 25× Fragmentation Buffer (Agilent Technologies, USA). The mixture was then heated at 60°C for 30 min, and 25 µl 2× GE Hybridization buffer (Agilent Technologies, USA) was added to dilute the labelled cRNA. Fifty µl of hybridization solution was dispensed into the gasket slide and assembled to the Mouse LncRNA Array v2.0 (8×60 K, Arraystar Inc., Rockville, USA) microarray slide. The slides were incubated for 17 hours at 65°C in an Agilent Hybridization Oven. The hybridized arrays were washed, fixed and scanned on the Agilent DNA Microarray Scanner (part number G2505C). LncRNAs (31,423) and 25,376 coding (mRNA) transcripts which were collected from the authoritative data sources including RefSeq, UCSC Known genes, Ensembl and many related literatures could be detected. Agilent Feature Extraction software (version 11.0.1.1) was used to analyze the acquired array images.

### MicroRNA microarray

LNA-modified oligonucleotide (Exiqon, Denmark) probes for mouse miRNAs annotated in miRBase version 16.0 were used in the microarray that was carried out in our laboratory. Total RNA (1 µg) from 4 separate experiments (n = 4) was pooled for each time point (Day 2, Day 4, Day 6, Day 8, 0 hr OGD) and 3′-end –labelled with Hy3 dye using the miRCURY LNA Power Labelling Kit (Exiqon, Denmark). The labelled RNA was hybridized on miRCURY LNA Arrays, using MAUI hybridization system for 17 hours at 56°C. The hybridized arrays were washed, fixed and scanned on InnoScan 700 microarray scanner (Innopsys, Carbonne, France). The digitized images were captured and analysed by MAPIX®4.5 (Innopsys, Carbonne, France) microarray image analysis software.

### Pathway and gene ontology and target prediction analyses

Gene ontology (GO) analysis for differentially expressed mRNA (log2 transformed, Signal Log Ratio (SLR)>1 or SLR<−1 relative to Day 2) was performed in the standard enrichment computation method using the Agilent GeneSpring GX software (version 11.5.1). For associated genes of lncRNAs (SLR>1 or SLR<−1 relative to Day 2), overrepresented GO biological processes were assigned using FuncAssociate with *p<0.05* [44]. Overrepresented GO biological processes for differentially expressed miRNAs (SLR>0.6 or SLR<−0.6 relative to Day 2) were assigned using Starbase with *p<0.05* [45]. Genes that had differentially expressed mRNA as well as lncRNA associated with them were selected for pathway enrichment analysis using the web-accessible program, Database for Annotation, Visualization and Integrated Discovery (DAVID) version 6.7 [46], [47]. Both TargetScan (http://www.targetscan.org/) and microRNA.org (http://www.microrna.org/microrna/home.do) were utilised to predict the targets of the selected microRNAs [48], [49]. RNAhybrid (http://bibiserv.techfak.uni-bielefeld.de/rnahybrid/) was used for microRNA target prediction for lncRNA. This tool determined the minimum free energy hybridisation for a long and short RNA, to predict the targets for ncRNAs [50].

### Statistical analyses

The Cy3 signal intensity average for the both lncRNA and mRNA microarrays were 500±200. For miRNA microarray, the Hy3 signal intensity average was 1600±200. For mRNA and lncRNA data analyses, quantile normalization was carried out for each chip and subsequent data processing was performed using the GeneSpring GX v11.5.1 software package (Agilent Technologies, USA). For miRNA data analysis, background-subtracted mean signal intensity of 300 was selected as a threshold for inclusion of significantly detected miRNAs followed by normalization against a group of endogenous controls for each chip. For comparison of mRNA, lncRNA and miRNA profiles, the above (independently normalized data) was subjected to a second normalization where data for maturing neurons was normalized to day 2 while data for neurons subjected to OGD was normalized to 0 hr control. The final normalized signal intensity was log2 transformed (Signal Log Ratio, SLR). Hierarchical clustering plots for differentially expressed mRNAs, lncRNAs and miRNAs were generated using TIGR multiple experimental viewer software [51]. One sample *t-test* was used to calculate the *p-value* from 2 replicates for comparison of expression during maturation (Day 6 and Day 8) and OGD (2 hr and 4 hr OGD). Day 2 and 0 hr OGD were used as the µ values for maturation and OGD respectively. Student’s *t-test* was used to determine statistical difference between 2 hr and 4 hr OGD. Pearson correlation coefficient (*R*) was determined between the SLR and days of maturation or hours of exposure to OGD. All the microarray data described in this study have been deposited in the NCBI Gene Expression Omnibus (GEO) and can be retrieved under accession number GSE44834.

## Results

### Maturation of cortical-neuron

For an *in vitro* model of neuronal maturation, pure cortical neuronal cultures were necessary. Primary neuronal cultures were established from embryos of E14, E15 and E16 pregnant Swiss albino mice according to the published protocols [25]–[28], in order to determine the optimal embryonic day to obtain pure neurons. Immunostaining with the neuronal markers, microtubule-associated protein 2 (MAP2) and neuronal nuclei (NeuN) [31], a neuronal progenitor marker, (SRY (sex determining region Y)-box 2 (Sox2) [32], microglial marker (CD11b) [33], oligodendrocyte marker (O4) [34] and astrocyte marker (glial fibrillary acidic protein, GFAP) [35] was carried out. Cultures derived from all embryos yielded neurons which matured from Day 2 to Day 8. This was evident from the increased MAP2 staining that showed the presence of neurite outgrowth (Figure 1, Figure S1A, Figure S1B). The positive staining for increased MAP2 signals were also accompanied by increasing NeuN staining from Day 2 to Day 8 indicating the presence of post-mitotic neurons particularly on the later days, Days 6 and 8 in culture (Figure 1, Figure S1A, Figure S1B). E14 cultures stained for Sox2 on Day 2 (Figure S1A), indicating presence of neuronal progenitors but these were absent in E15 cultures. The E15 cultures also showed absence of CD11b, O4 and GFAP staining, confirming that glial cells were not present as well (Figure 1). Though the E16 cultures were absent for Sox2 it showed staining for GFAP on Days 6 and 8, indicating presence of astrocytes in the cultures (Figure S1B). Hence, E15 derived neuronal cultures which displayed increased staining for MAP2 and NeuN from Day 2 to Day 8, reflective of neuronal maturation, but absence of Sox2, CD11b, O4 and GFAP staining, were found to be purely post-mitotic cultures, and were chosen for the study. Early neurite outgrowth was observed on Day 2 (Figure 1) which became more extensive with maturation on Day 4 (Figure 1). Intense staining with MAP2 on days 6 and 8 indicated establishment of a mature neuronal network.

Qualitative analysis of the E15 cultures were determined by using neuronal specific markers MAP2 and NeuN. Immunostaining images confirmed that all of the cells in our E15 cultures were of the neuronal cell type (Figure 1). MAP2 also served as a positive marker for neuronal maturation (Figure 1) [31]. This was also supported by quantitative measurements of miRNA abundance. We found that miR-124, a neuron specific miRNA, was highly expressed (Day 8 C_T_ = 21.0±0.0 compared to NTC C_T_ = Undetermined, *p<0.01*; Table 1) and the C_T_ values obtained for the astrocyte-enriched miR-143 and glia-enriched miR-223 were determined to be non-significant as compared to the no template control (NTC; Table 1) [37]. These results suggested that contamination from astrocytes and glial cells was negligible or absent in our neuronal cultures.

### Transcriptome of maturing neurons

**Figure 2:**
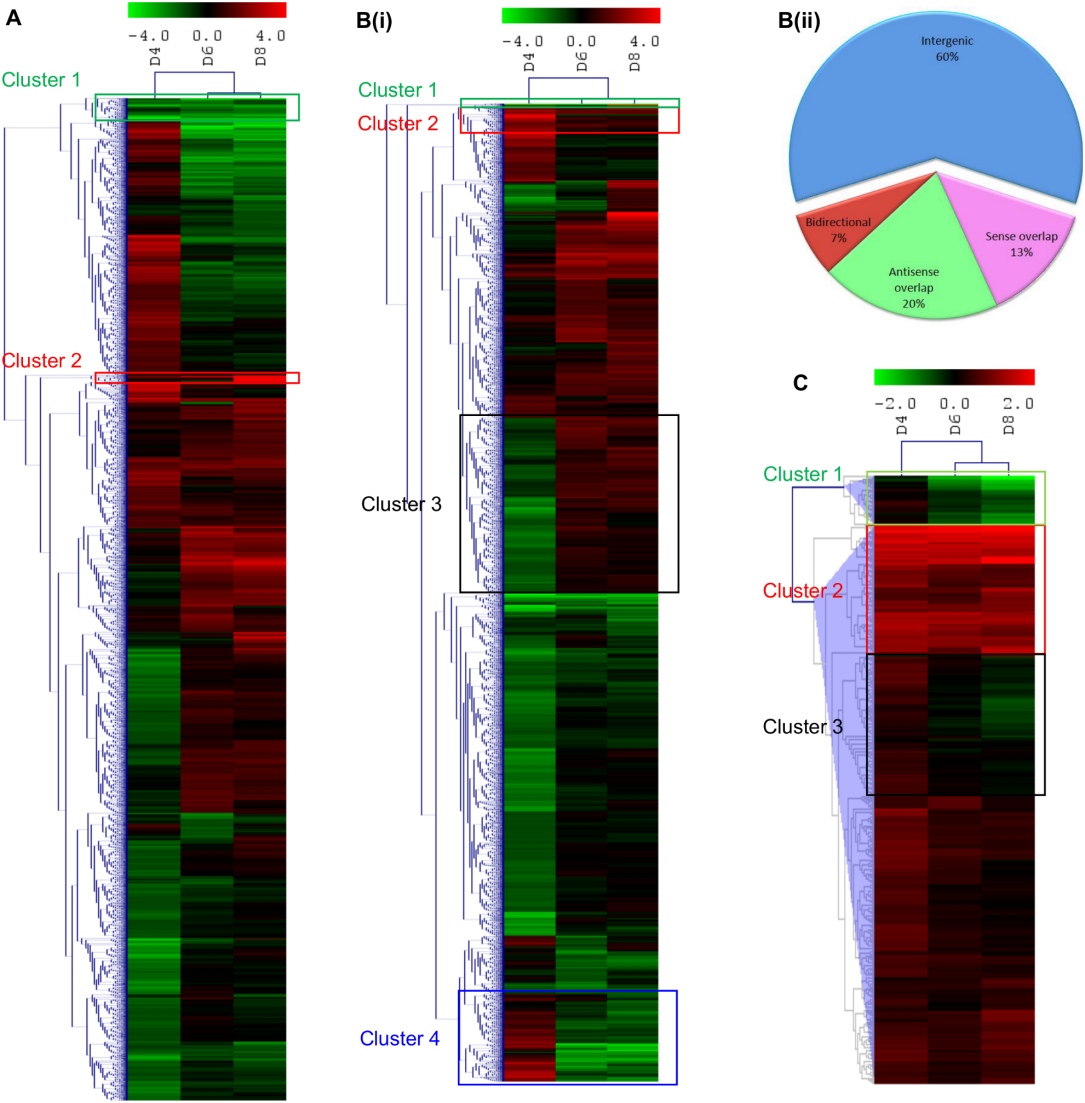
Transcriptome of maturing neurons. (**A**) Hierarchical clustering analyses of mRNAs in maturing cortical neurons. Clusters of highly down- and up-regulated mRNAs were identified. **Bi)** lncRNAs in maturing neurons. Clusters of highly down- and up-regulated lncRNAs with their orientation to the genome or proximal gene loci were identified. (**Bii**) Subgroup analyses of altered lncRNAs in relation to their nearby coding genes. (**C**) miRNAs in maturing cortical neurons. Clusters of highly down- and up-regulated miRNAs were identified. Total RNA from 4 separate experiments (n = 4) carried out in triplicates were pooled for each time points. The microarray analyses were carried out for each time point on the pooled RNA. The average signal intensities were 369.03, 501.12, 429.58 and 455.03 for the lncRNA and mRNA microarray for Day 2, Day 4, Day 6 and Day 8 respectively. For miRNA microarray, the average signal intensities were 1673.43, 1783.01, 1385.10 and 1698.38 for Day 2, Day 4, Day 6 and Day 8 respectively. Hierarchical clusters were constructed out using average linkage and Euclidean distance as the similarity measure. Green rectangle indicates downregulation and red, upregulation.

Maturing neurons were used to study the changes in the transcriptome which would favour neuronal development and survival, hence serving as a pro-survival model. Changes in the transcriptome over the 8 days were determined *via* RNA expression profiling. Expression patterns of mRNAs, lncRNAs and miRNAs during maturation were determined by normalizing gene expression to Day 2. Independent hierarchical clustering (HCL) analysis of all three sets of data showed that day 4 was clustered further from days 6 and 8, possibly reflecting a more stable and distinct neuronal network on the latter days (Figure 2A, 2B(i), 2C). The similar HCL between the 3 separate entities also highlighted the existence of the tight regulatory interaction among them.

Of the 14213 mRNA transcripts detected through profiling, 6965 (49.0%) of them exhibited differential gene expression (SLR>1 or SLR<−1; Figure 2A). For instance, two clusters of highly down- and up-regulated genes were observed (Figure S2A).

Profiling of lncRNAs detected 15715 transcripts and approximately 47.4% (7455) of these showed differential expression (SLR>1 or SLR<−1) on at least one of the days as compared to Day 2 (Figure 2B(i), Figure S2B, Table S3). About 2.3% of these differentially expressed lncRNAs were derived from ultraconserved segments (100% identity with no insertions or deletions) between orthologous regions of the human, rat, and mouse genomes. Among the altered lncRNAs, 60% of them were intergenic. The remaining 2993 lncRNAs were associated with known genes in either an antisense (20%) or sense overlap (13%) or in a bidirectional (7%; head to head to a coding transcript within 1000 bp) manner (Figure 2B(ii)).

Of the 1040 miRNA probes, 395 (38.0%) were detected on our miRNA profiling. Two distinct clusters of down- (Clusters 1 and 3; Figure S2C) and up-regulated miRNAs (Cluster 2; Figure S2C) were identified in the mature neurons (Figure 2C).

Independent Gene Ontology (GO) analysis on the transcriptome data identified dendritic morphogenesis and axonogenesis, processes associated with neuronal differentiation, as being regulated by altered mRNAs as well as both lncRNAs and miRNAs (Figure S3). Processes other than those directly associated with neuronal differentiation were also observed to be regulated by the 3 separate entities (Figures S3A, S3B, S3C). This could possibly be due to their ability to participate indirectly in other biological processes.

### Identification of pathways and genes essential for neuron development and survival

**Figure 3:**
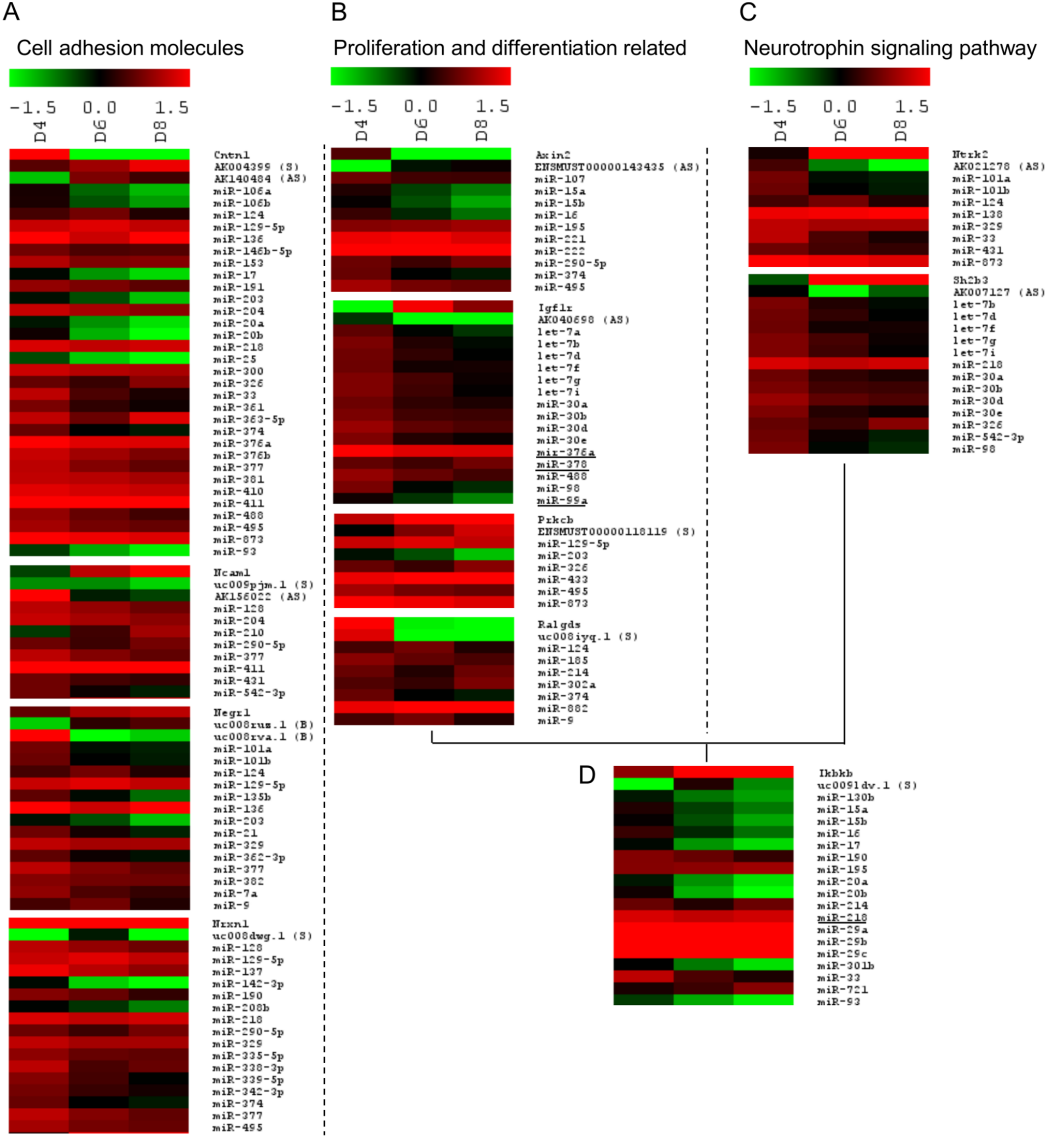
Differentially expressed RNA transcriptome associated with (A) cell adhesion molecules regulation, (B) proliferation and differentiation pathways, (C) neurotrophin signalling pathway, (D) differentially expressed *Ikbkb* and its associated ncRNAs as determined by microarray (pooled RNA from 4 separate experiments for each time point). mRNA expression of the gene is indicated followed by the expression of the associated lncRNA (S: sense overlap; AS: antisense overlap; B: bidirectional). Differentially expressed miRNAs that are predicted to target the gene (TargetScan and microRNA.org) are indicated. Validated miRNAs are underlined. Expression is indicated in SLR relative to Day 2. Red, indicates upregulation and green, downregulation.

To elucidate genes that were regulated by both ncRNAs, we adopted a systematic approach to first shortlist genes with both differentially expressed mRNAs and lncRNAs associated with them. Subsequent pathway analysis on the shortlisted genes revealed proliferation and differentiation related pathways, cell adhesion molecules and neurotrophin signalling to be over-represented (Table 2). Of these, 23 genes were predicted targets of the differentially expressed miRNAs (Table 2 shows the 23 genes in bold, the altered miRNAs predicted to target the mRNA of these genes are listed in Table S4). Next, we wanted to identify the genes that are crucial in maintaining the mature neuronal phenotype. We identified 11 out of the 23 genes to be differentially expressed on days 6 and 8. These 11 genes were also found to be implicated in the three pathways identified in Table 2 (Figure 3).

**Table 2:** Pathways implicated during neuronal maturation.

DESCRIPTION	*p-value*	GENES
**Proliferation and differentiation related pathways**	*0.005*	***Abl1*** *, Araf, * ***Axin2*** *, Brca2, Cdh1, Ctnna2, * ***E2f2*** *, Fgf12, * ***Fgfr1*** *, Gli2, * ***Igf1r*** *, * ***Ikbkb*** *, Ikbkg, * ***Itgb1*** *, * ***Kit*** *, Pax8, Plcg1, * ***Prkcb*** *, Ptch1, * ***Ptgs2*** *, Pik3cd, * ***Ralgds*** *, Tcf7l1, Wnt11*
**Cell adhesion molecules**	*0.02*	*Cd40, Cd8b1, Cdh1, * ***Cdh4*** *, * ***Cntn1*** *, * ***F11r*** *, H2-d1, * ***Itgb1*** *, Madcam1, * ***Ncam1*** *, * ***Negr1*** *, * ***Nrxn1*** *, * ***Nrxn3***
**Neurotrophin signalling pathway**	*0.015*	***Abl1*** *, Calm1, * ***Camk2d*** *, * ***Ikbkb*** *, * ***Ntrk2*** *, Pik3cd, Plcg1, * ***Rapgef1*** *, Sh2b1, * ***Sh2b3*** *, * ***Sort1*** *, Trp73*
**Prostate cancer**	*0.01*	*Araf, Creb3l2, E2f2, Fgfr1, Igf1r, Ikbkb, Ikbkg, Pdgfc, Pik3cd, Tcf7l1*
**Glioma**	*0.013*	*Araf, Calm1, Camk2d, E2f2, Igf1r, Pik3cd, Plcg1, Prkcb*

Genes identified had differentially expressed mRNAs and lncRNAs associated with them. mRNA of genes in bold were predicted to be targets of the altered miRNAs as shown in Table S4.

Genes identified in the proliferation and differentiation related pathways (Table 2) regulate cell cycle arrest that is required for neuronal differentiation as well as axonal outgrowth and cell survival. Of these, *Axin2, Igf1r, Ikbkb, Prkcb* and *Ralgds* had differentially expressed mRNAs and lncRNAs associated with them in mature neurons (Days 6 and 8) and were also predicted targets of the differentially expressed miRNAs (Figure 3). A decreased expression of *Axin2* and *Ralgds* and up-regulation of *Igf1r*, *Ikbkb,* and *Prkcb* mRNAs were observed in the mature neuronal phenotype (Days 6 and 8; Figure 3B, 3D). LncRNAs associated with *Axin2* (ENSMUST00000143435), *Igf1r (*AK040698) and *Ikbkb* (uc009ldv.1) showed a reciprocal expression profile with their mRNA. mRNA-lncRNA pairs of *Prkcb* (ENSMUST00000118119) and *Ralgds* (uc008iyq.1), however, showed a similar expression profile.

Amongst the cell adhesion molecules *Cntn1, Ncam1, Negr1* and *Nrxn1* had differentially expressed mRNAs and lncRNAs associated with them and were also predicted to be targeted by the altered miRNAs (Figure 3A). Expression of *Ncam1*, *Negr1* and *Nrxn1* was upregulated and expression of *Cntn1* was downregulated upon maturation (Days 6 and 8). *Cntn1*, *Ncam1*, *Negr1* and *Nrxn1* mRNA-lncRNA pairs displayed an inverse relationship (*Cntn1*: AK004399 and AK140484; *Ncam1*: uc009pjm.1 and AK156022; *Negr1*: uc008rva.1; *Nrxn1*: uc008dwg.1). *Negr1* had another lncRNA associated with it which showed a similar expression (uc008ruz.1) with its mRNA.

In the neurotrophin signalling pathway *Ikbkb* (common to the proliferation and differentiation pathway), *Ntrk2* and *Sh2b3* had differentially expressed mRNAs and lncRNAs associated with them and were predicted to be co-regulated by the altered miRNAs. Elevated levels of *Ntrk2* and *Sh2b3* mRNA transcripts were observed in mature neuronal phenotype with a reciprocal expression profile to their respective lncRNAs (Days 6 and 8; Figure 3C).

Based on these observations, we postulated that regulation of these 11 genes (*Axin2, Igf1r*, *Ikbkb, Prkcb, Ralgds, Cntn1, Ncam1, Negr1, Nrxn1, Ntrk2* and *Sh2b3*) by both lncRNAs and miRNAs could be implicated in neuronal maturation. The expression of the 11 mRNAs, 1 randomly selected lncRNA per gene and miRNAs was validated by qPCR in maturing neurons on Days 2, 4, 6, 8 as well as more mature neurons on Day 14 (Tables S5 and S6). Expression validation showed that the expression profile (Pearson’s correlation coefficient, *R*) on the first 8 days (*R_array_*) was consistent up to Day 14 (*R_qPCR_*). In view of this, subsequent analysis was based on the first 8 days. To further characterize how lncRNAs regulate these genes upon ischemia, their expression was determined in neurons subjected to an *in vitro* model of ischemic-reperfusion injury (OGD).

### mRNA-IncRNA expression patterns in ischemic injury

**Figure 4:**
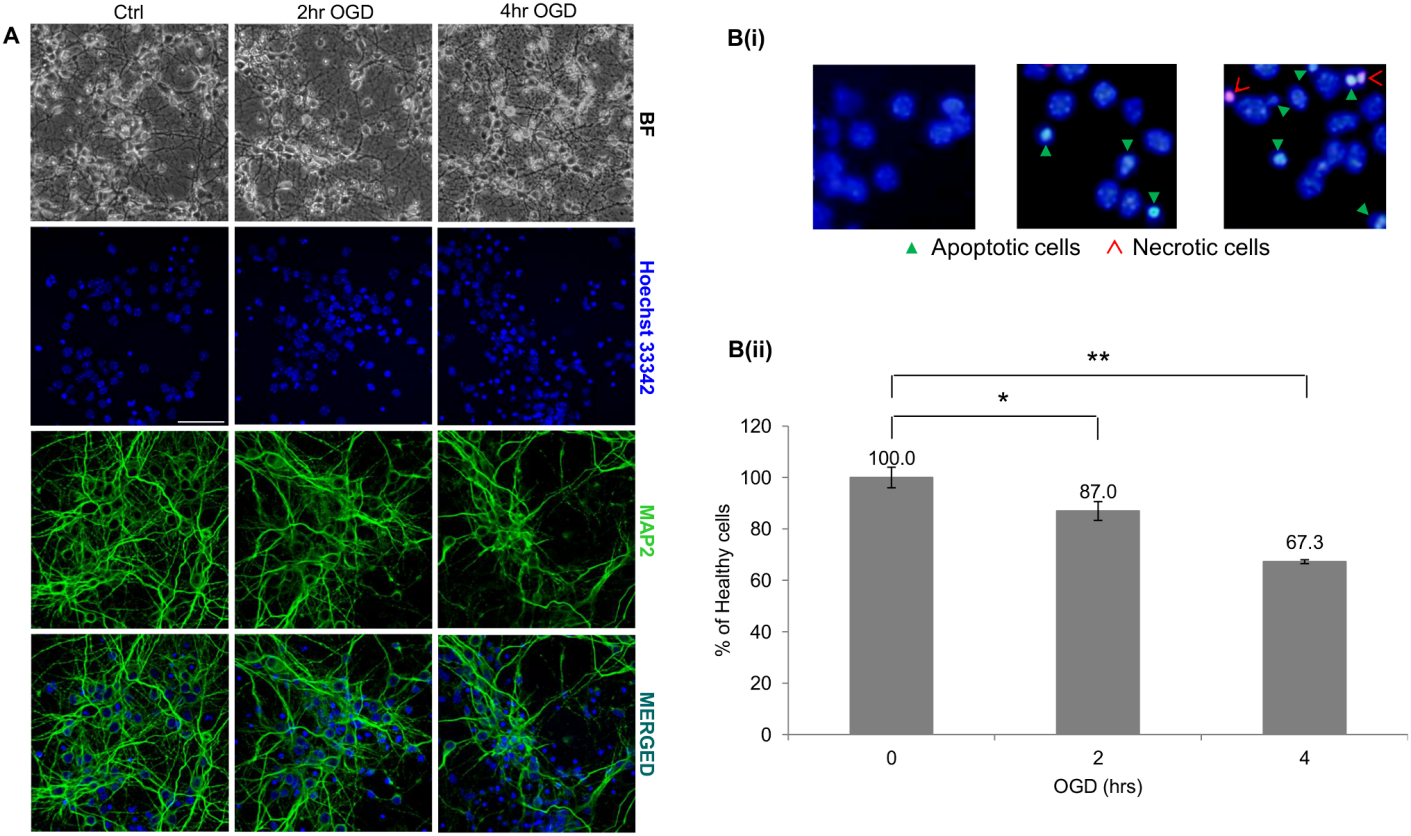
Primary neurons subjected to oxygen and glucose deprivation (OGD). (**A**) Bright-field immunostained images illustrating cell death in healthy neuronal cultures subjected to OGD. Cells subjected to increasing ischemic injury displayed degenerated neurites. BF-Bright-field; Green-MAP2; Blue-Hoechst 33342. Scale bar represents 50 µm. (**Bi**) Primary neuronal cultures subjected to OGD were stained with the nuclear stain, Hoechst 33342 (blue), and necrotic cells marker, Ethidium homodimer III (red). Exposure to OGD showed an increase in apoptotic cells, indicated by condensed nuclei (green arrow heads) and necrotic cells (red open arrow heads). (**Bii**) Healthy cells were expressed as a percentage (mean ± SD) of the total cells. All experiments were carried out n = 4 and in triplicates. Statistically significant differences were tested using the Student’s *t-test* (*p<0.05, **p<0.01).

Primary mouse cortical neurons, on the day 6 of culture were subjected to OGD (2, 4 hr) followed by 24 hr reperfusion. OGD resulted in degenerated neurites (Figure 4A) and the percentage of healthy cells significantly decreased from 2 hr to 4 hr of OGD (Figures 4Bi and 4Bii). Expression of mRNAs and lncRNAs associated with the genes identified earlier were verified in these samples to identify the roles of these transcripts in neuronal survival (maturation) and cell death (OGD).

The relative changes in mRNA and lncRNA expression for the 11 genes during ischemic injury is shown in Table 3. Next, we performed Pearson’s correlation on the 11 genes based on their mRNA and lncRNA expression in the two models; maturation and OGD. A reversal in direction (+/− or −/+) of the Pearson’s correlation coefficient was observed from maturation to ischemic injury for the mRNA and lncRNA expression associated with most of the genes. For instance, *Negr1* mRNA (NM_001039094) showed a Pearson’s correlation coefficient of 0.96 during maturation which was reversed to −0.99 during ischemic injury. This indicated an opposite expression profile upon ischemic injury as compared to during maturation (Table 3).

**Table 3:** Expression of mRNA and lncRNAs associated with selected genes identified in the proliferation and differentiation associated pathway, cell adhesion molecules and neurotrophin signalling pathway during ischemic injury.

Gene	mRNA	Genomic orientation of lncRNA		Expression in ischemic injury			Pearson’s correlation coefficient (*R*)					mRNA-lncRNA relationship
	lncRNA			2 hr OGD		4 hr OGD	Maturation			Ischemic injury		
**Proliferation and differentiation related pathway**												
***Axin2***	NM_015732			0.03±0.03		1.54±0.33^##^	−0.83**			0.88		
	ENSMUST00000143435	antisense overlap, intron-exon		−0.46±0.06		−0.26±0.03^#^	0.28			−0.55*		Inverse
***Igf1r***	NM_010513			0.07±0.01		0.25±0.03^##^	0.54*			0.97		
	AK040698	antisense overlap, intron		1.34±0.31		1.31±0.26	−0.97*			0.86**		Inconclusive
***Ikbkb***	NM_010546			−0.49±0.09		0.10±0.01^##^	0.87**			0.16		
	uc009ldv.1	sense overlap, promoter		−2.05±2.06		−1.22±0.38	−0.04			−0.59*		Inconclusive
***Prkcb***	NM_008855			−0.89±0.13		−0.23±0.03^##^	0.95**			−0.25		
	ENSMUST00000118119	sense overlap, intron		−0.82±0.09		−0.49±0.05^##^	0.94*			−0.59*		Synergistic
***Ralgds***	NM_009058			0.12±0.02		−0.25±0.05^##^	−0.64**			−0.65		
	uc008iyq.1	sense overlap, intron-exon		1.23±0.15		0.41±0.06^##^	−0.73**			0.32		Inconclusive
**Cell adhesion molecules**												
***Cntn1***	NM_001159648		0.34±0.06		0.39±0.06			-0.78*	0.91**			
	AK004399	sense overlap, 3'UTR	−0.33±0.03		−0.59±0.06^##^			0.99*	−1.00*		Inverse	
	AK140484	antisense overlap, intron	0.09±0.01		−0.33±0.04^##^			0.47*	−0.75		Inverse	
***Ncam1***	NM_010875		−1.38±0.21		−0.35±0.04^##^			0.89*	−0.25			
	uc009pjm.1	sense overlap, promoter	−0.23±0.02		0.00±0.10^##^			−0.92*	−0.01		Inconclusive	
	AK156022	antisense overlap, intron	1.34±0.13		0.69±0.07^##^			−0.41	0.52*		Inverse	
***Negr1***	NM_001039094		−0.39±0.04		−0.60±0.06^#^			0.96**	−0.99*			
	uc008ruz.1	Bidirectional	−0.98±0.13		−0.70±0.08			0.50*	−0.70*		Synergistic	
	uc008rva.1	Bidirectional	0.98±0.24		1.15±0.24			−0.53*	0.93**		inverse	
***Nrxn1***	NM_020252		−0.93±0.18		−0.14±0.02^##^			0.21**	−0.14			
	uc008dwg.1	sense overlap, intron	0.08±0.02		0.35±0.06^##^			−0.48	0.95		Inverse	
**Neurotrophin signalling pathway**												
***Ntrk2***	NM_008745		−0.36±0.04		−0.25±0.02^#^			0.96**	−0.68*			
	AK021278	antisense overlap, intron	0.16±0.02		−0.49±0.05^##^			−0.87**	−0.72		Inconclusive	
***Sh2b3***	NM_008507		−1.50±0.34		−1.77±0.42			0.84**	−0.93**			
	AK007127	antisense overlap, first intron	−0.06±0.02		0.37±0.07^##^			−0.53*	0.79		Inverse	

Reqseq number for the mRNA of each gene and the sequence number of the associated lncRNA are indicated. The average signal intensities were 618.09, 388.01 and 644.28 for the lncRNA and mRNA microarray carried out for 0 hr, 2 hr and 4 hr OGD respectively. Expression is shown in SLR ± SD relative to Day 2. Pearson’s correlation coefficient (*R*) was computed between SLR and days after maturation or hours of exposure to OGD ischemic injury. One sample *t-test* was used to calculate the *p-value* from 2 replicates for comparison of expression during maturation (Day 6 and Day 8) and OGD (2 hr and 4 hr OGD). Day 2 and 0 hr OGD were used as the µ values for maturation and OGD respectively (*p<0.10, **p<0.05). Student’s *t-test* was used to determine statistical difference between 2 hr and 4 hr OGD (^#^ *p<0.10*, ^##^ *p<0.05*). The inferred relationship between the mRNA-lncRNA expression from maturation and ischemic injury as well as genomic orientation of the associated lncRNA is also indicated.

**Figure 5:**
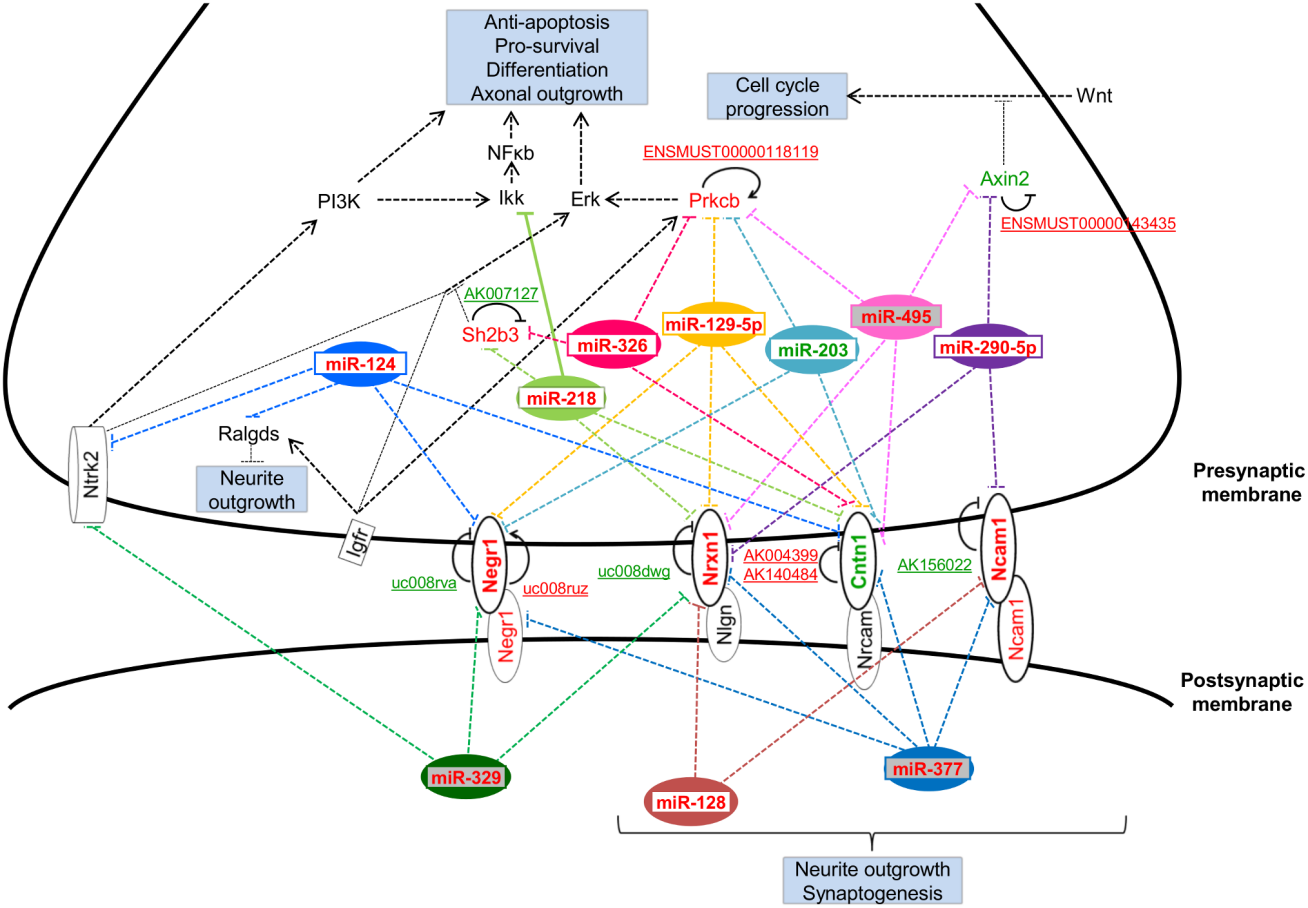
Overview of integrated network of genes regulated by both lncRNAs and miRNAs, that are crucial for precise neuronal development. Upregulated mRNAs/lncRNAs/miRNAs are shown in red font. Green font represents downregulated mRNAs/lncRNAs/miRNAs. LncRNAs associated with the gene are underlined. Black solid feedback arrows (→) on the gene indicate lncRNAs associated with the gene with synergistic expression. Black inhibitory arrows (⊥) indicate lncRNAs associated with the gene with reciprocal expression. Ten differentially expressed miRNAs targeting the respective genes are indicated. Solid lines (–) emerging from the miRNAs indicate validated targets. Dashed lines (----) indicate predicted targets as determined by TargetScan and microRNA.org. miRNAs shaded in grey (miR-329, -377, -495) belong to the miR-379-410 cluster.

Further interrogation of Pearson’s correlation coefficient of the mRNA-lncRNA pairs for each gene in the maturation and ischemic models was used to determine the relationship between the two entities (Table 3). Same direction (+/+ or −/−) of Pearson’s correlation coefficient for the mRNA-lncRNA in the 2 models was defined as a synergistic relationship between the mRNA and its associated lncRNA whereas an opposite direction (+/− or −/+) suggested an inverse relationship. For example, the *Prkcb* mRNA-lncRNA pair (sense overlap) in the proliferation and differentiation related pathway showed a synergistic relationship whereas the *Axin2* mRNA-lncRNA pair (antisense overlap orientation) in the same pathway was inversely related (Table 3). Furthermore, cell adhesion molecules such as *Cntn1*, *Ncam1*, *Negr1* and *Nrxn1* as well as *Sh2b3* in the neurotrophin signalling pathway displayed a mainly inverse relationship between mRNA and antisense lncRNA gene pairs. These findings seem to indicate a predominant reciprocal relationship between antisense lncRNAs and their associated mRNA. Among the 11 genes, we were able to observe a distinct inverse or synergistic expression between the mRNA and lncRNA of 7 genes namely, *Axin2, Prkcb*, *Cntn1*, *Ncam1*, *Negr1*, *Nrxn1*, *Sh2b3* (Table 3). Hence, the next step was to determine the miRNAs that could be regulating these 7 genes along with their respective lncRNAs (Figure 5).

### Regulation of axonogenesis and dendritogenesis by lncRNAs and miRNAs

Using the list of miRNAs altered during neuronal maturation (Figure 3), we mapped those that were predicted or validated to target 2 or more of the 7 genes. A total of 10 miRNAs, miR-124, miR-128, -129-5p, -203, -218, -290-5p, -326, -329, -377 and -495 were identified. An overview of the intricate regulatory network of lncRNAs and miRNAs over the 7 genes is shown in Figure 4. Interestingly, we observed possible regulation of cell adhesion molecules *Cntn1, Ncam1, Negr1* and *Nrxn1* which are responsible for neurite outgrowth and synaptogenesis (Figure 5, genes in bold).

### Quantitation of *Ncam1* and *Negr1* associated mRNAs, IncRNAs and miRNA

**Figure 6:**
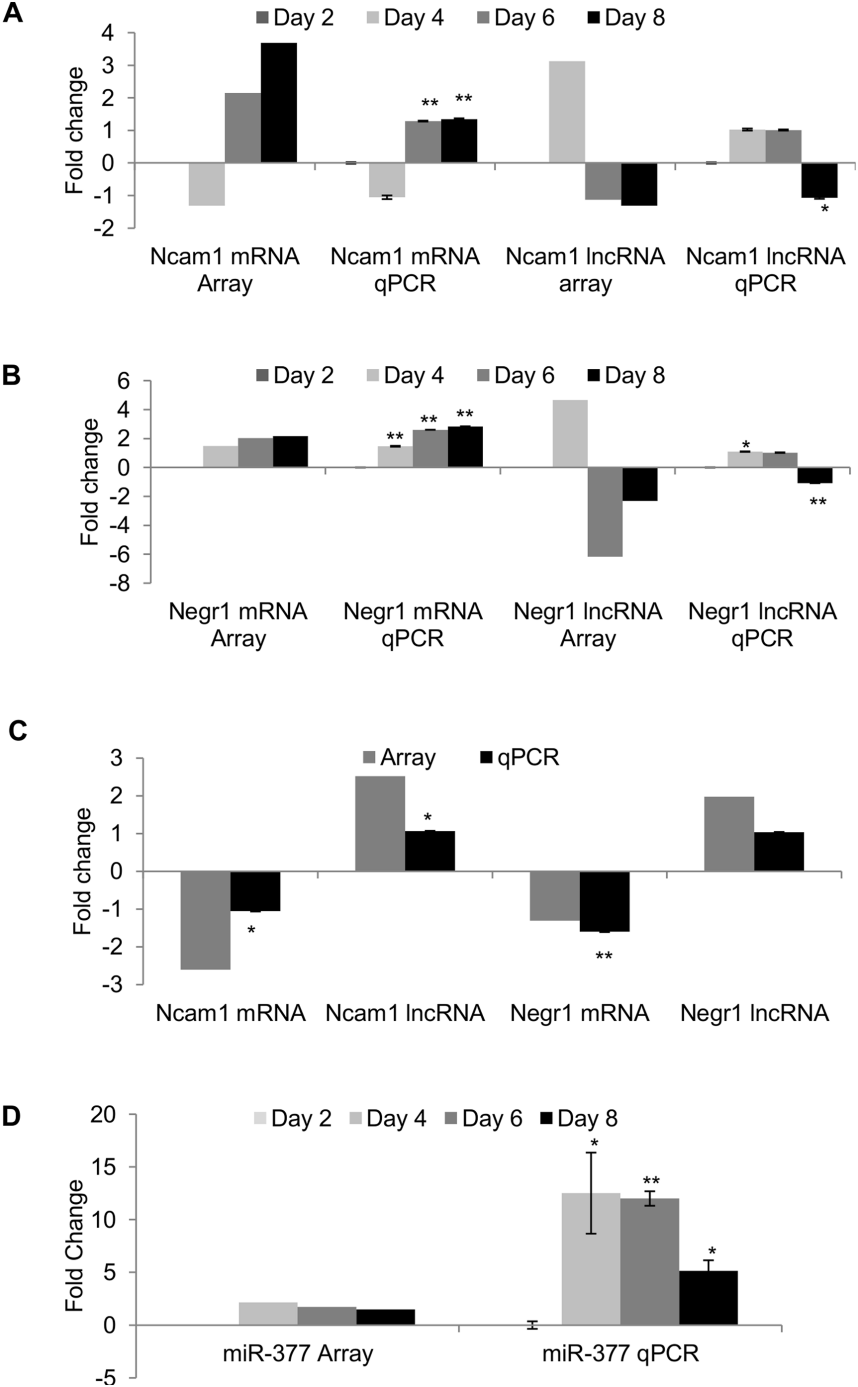
Validation and quantification of mRNA, lncRNA and miRNA expression by qPCR. (**A**) *Ncam1* mRNA (NM_010875) and lncRNA (AK156022), (**B**) *Negr1* mRNA (NM_001039094) and lncRNA (uc008rva.1), (**C**) *Ncam1* and *Negr1* mRNA-lncRNA pairs in neurons subjected to 2 hr OGD. (**D**) Stem-loop PCR quantification of miR-377 in maturing neurons. Expression of GAPDH was used as a control/housekeeping gene to normalize mRNA, lncRNA and miRNA expression. Statistically significant differences were tested using the Student’s *t-test* (*p<0.05, **p<0.01).

Of the 10 miRNAs, only miR-377 was predicted to target both *Ncam1* and *Negr1* and was therefore included in our validation study. Furthermore, recent studies have reported that both these genes promote neurite outgrowth as well as synapse maturation [52], [53]. Expression of these mRNA-lncRNA pairs as well as miRNA correlated to the microarray data analyses (Figure 6).

## Discussion

Non-coding RNAs are emerging as critical determinants and regulators of neuronal development not only by modulating global gene expression but also by regulating gene expression of their neighbouring or associated genes [54]–[57]. These non-coding RNAs are being widely studied as competing endogenous RNA (ceRNA), which govern the regulatory roles on genome wide gene expression [58]. In our study, we characterized the expression of mRNAs, lncRNAs associated with known protein-coding genes and miRNAs during neuronal differentiation and ischemic condition. Primary neuronal cultures were established from embryos of E15 pregnant Swiss albino mice and maintained in Neurobasal medium supplemented with B27 to ensure selective growth of cortical neurons (Figure 1) [25], [30]. We could obtain >99% neuronal (as determined by MAP2 and NeuN staining) cultures as reported by Valerio et al (2006) [36] and Yamasaki et al (2003) [28]. We also showed the absence of Sox2, CD11b, O4 and GFAP staining in our E15 neuronal cultures, indicating presence of purely post-mitotic neurons (Figure 1). Hence, expression studies on these cultures represent the expression changes associated with maturation of neurons similar to studies carried out by Valerio et al [36] and Chen et al [59]. The RNA expression profiles reported thus reflect changes in dendritic or axonal gene expression in neurons. Furthermore, as per our observation, the shortlisted genes such as *Negr1* and *Ncam1* have also been reported to be important in neuronal development [52], [60], [61]. Analyses of the expression profiles of both the mRNAs and lncRNAs revealed extensive regulation of genes (Figure 2A, Figure S2A) by lncRNAs (Figure 2B(i), Figure S2B, Table S3) during neuronal differentiation.

In this study, genes that were differentially expressed as well as had altered lncRNA expression associated with their locus were mapped to proliferation and differentiation associated pathways that are involved in cell cycle processes, cell adhesion molecules and neurotrophin signalling (Table 2). A large number of the genes identified in these pathways showed similar expression profiles between the miRNAs and their respective target mRNAs. This is unexpected as given the role of miRNAs as post-transcriptional regulators, an inverse expression profile is usually more likely between miRNAs and mRNAs [10]. Therefore, we proposed that the associated lncRNAs could be providing another level of modulation. The function of miRNAs as translational inhibitors is well-established. However, the mechanisms by which lncRNAs regulate gene expression are more diverse and not fully elucidated. Therefore, we proceeded to validate the expression of the 11 mRNA-lncRNA pairs (*Axin2, Igf1r*, *Ikbkb, Prkcb, Ralgds, Cntn1, Ncam1, Negr1, Nrxn1, Ntrk2* and *Sh2b3*) that is altered during neuronal maturation (Figure 3), in an ischemic injury model (neuronal). Comparison of the mRNA and lncRNA expression profiles between a survival (neuronal maturation) model and an ischemic injury (OGD) model allowed us to determine the potential role of the lncRNAs in regulating the expression pattern of their respective mRNAs.

Expression profiles of *Axin2, Igf1r, Ikbkb, Prkcb, Ralgds, Cntn1, Ncam1, Negr1, Nrxn1, Ntrk2* and *Sh2b3* associated mRNAs and lncRNAs during ischemic injury could characterize the exact relationship between 7 mRNA-lncRNA targets (Table 3). An inverse expression of these genes (*Axin2*, *Prkcb*, *Cntn1*, *Ncam1*, *Negr1*, *Nrxn1*, *Sh2b3*) was observed upon ischemic insult (Table 3). Similarly, *NB-3*, a cell adhesion molecule expressed in neurons for axonal extension and neuronal survival, has been reported to be inversely regulated during ischemic injury, resulting in impaired neuronal survival and neurite outgrowth [18]. Moreover, the mRNA microarray data showed similar results to those from rodent ischemic stroke models (middle cerebral artery occlusion, MCAo), indicating our *in vitro* model of ischemic injury is reflective of the gene expression changes taking place in an *in vivo* ischemic injury model [15]. Our observations on the inverse regulation of genes crucial to neuronal function upon ischemic injury are hence consistent with previous reports [15], [18].

An inverse relationship was inferred between the *Axin2*, *Cntn1*, *Ncam1*, *Negr1*, *Nrxn1* and *Sh2b3* mRNA-lncRNA pairs while *Prkcb* showed a synergistic relationship between its mRNA-lncRNA pair. The distinct relationship between the lncRNA-mRNA pairs suggests a transcriptional or posttranscriptional regulatory function for lncRNAs in neuronal development. Consequentially, derailment of lncRNA expression upon ischemic injury may be a contributing factor to cell death.

Additionally, we also identified post-transcriptional regulation of the 7 lncRNA regulated genes (*Axin2*, *Cntn1*, *Ncam1*, *Negr1*, *Nrxn1*, *Prkcb*, *Sh2b3*) by miR-124, -128, -129-5p, -203, -218, -290-5p, -326, -329, -377 and miR-495. Among these, miR-124, -128, -129-5p, -218, -326, -329, -377 and -495 have been reported to exhibit brain specific or brain-enriched expression pattern [37], [62]–[66]. miR-329, -377 and -495 belong to the miR-379-410 cluster that modulates activity-dependent dendritogenesis and miR-495 also regulates expression of brain-derived neurotrophic factor (*BDNF*) [67], [68]. Interestingly, the cell adhesion molecules (*Cntn1*, *Ncam1*, *Negr1*, *Nrxn1*) were targeted by all these miRNAs with miR-377 specifically targeting only the 4 cell adhesion molecules (Figure 5).

Of these 4 cell adhesion molecules, *Ncam1* and *Negr1* mRNAs, implicated in neurite outgrowth, showed constitutive upregulation during maturation with downregulation of the associated lncRNAs (Figure 6) [52], [53], [69]. Increased expression of the cell adhesion molecules also coincided with extensive upregulation of neuronal-enriched miR-377 predicted to target these mRNAs (Figure 6). This observation is consistent with another study which suggested that miRNAs could be involved in neuronal homeostasis [70]. Interestingly, miR-377 is derived from the neuron-enriched miR-379-410 cluster. In response to increased neuronal activity, transcription of miR-134, another member of miR-379-410 cluster, is induced to promote neurite outgrowth [67]. Being in the same cluster, it is likely that miR-377 shares a similar function.

In this study, we identified regulation of a network of neuron-specific transcriptomes during neuronal maturation. The expression of the cell adhesion molecules, *Ncam1* and *Negr1* mRNAs during neuronal development could be modulated by lncRNAs and with the fine-tuning function of miR-377, results in precise neuronal maturation. Hence, this coordinated regulatory network could be the key to modulating neuronal homeostasis for precise neuronal development. Dysregulation of this delicate lncRNA-mRNA-miRNA network during ischemic insult (Table 3) appears to be a contributing factor to neuronal cell death.

## Supporting Information

Figure S1 **Maturation of (A) E14 and (B) E16 derived mouse primary cortical neuronal cultures.** Cultures were immunostained for neuronal markers (MAP2 and NeuN), a neuronal progenitor marker (Sox2), microglial marker (CD11b), oligodendrocyte marker (O4) and astrocyte marker (GFAP). Short neurites at the early stages of development (Day 2) gave rise to an extensive network of neurites by day 8. E14 cultures displayed presence of neuronal progenitors whereas E16 cultures showed presence of astrocytes. Scale bar represents 50 µm.
(PDF)

Figure S2 **Transcriptome of E15 derived primary cortical neuronal cultures (A) Differentially expressed genes in each cluster as identified in hierarchical clustering analysis of 6965 differentially expressed mRNAs (SLR>1) in maturing neurons (** **Figure 2A** **).** Genes showing −6<SLR<−1.5 (cluster 1), 3.5<SLR<7.5 (cluster 2) on Day 6 or Day 8 are shown to represent the cluster. Green indicates downregulation and red, upregulation. **(B) Differentially expressed lncRNAs in each cluster as identified in hierarchical clustering analysis of 7455 differentially expressed lncRNAs (SLR>1) in maturing neurons (** **Figure 2B** **(i)).** LncRNAs showing SLR = −8 (cluster 1), 2.4<SLR<3.4 (cluster 2), 1.3<SLR<2 (cluster 3), −4.5<SLR<−3 (Cluster 4) on day 6 or day 8 are shown to represent the cluster. Green indicates downregulation and red, upregulation. Genes associated with the lncRNAs in each cluster are stated in Table S3. **(C) Differentially expressed miRNAs in each cluster as identified in hierarchical clustering analysis of 395 miRNAs after background subtraction of signal intensity less than 300 in maturing cortical neurons (** **Figure 2C** **).** miRNAs showing −3<SLR<−0.3 (cluster 1), 1.2<SLR<2.2 (cluster 2), −0.1<SLR<−0.6 (Cluster 3) on day 6 or day 8 are shown to represent the cluster. Green indicates downregulation and red, upregulation.
(PDF)

Figure S3 **Biological processes associated with differentially expressed (A) mRNAs, (B) lncRNAs associated with genes in sense/antisense/bidirectional orientation, (C) miRNAs.** Green rectangle indicates fold enrichment for downregulated associated/predicted genes; red indicates fold enrichment of associated/predicted genes. (D) Biological processes which are common to the differentially expressed mRNAs, lncRNAs, and miRNAs.
(PDF)

Table S1 **Specific primers for mRNAs and lncRNAs of 11 shortlisted genes.**
(PDF)

Table S2 **MIQE checklist for authors, reviewers and editors.** Essential (E) and desirable information (D) has been made available in this table. Further details have been described in the main manuscript.
(PDF)

Table S3 **Associated gene names of lncRNAs for each cluster indicated in Figure S2B.**
(PDF)

Table S4 **List of 23 differentially expressed mRNAs in the top 3 pathways and the differentially expressed miRNAs that were predicted to target them.**
(PDF)

Table S5 **Validation and quantification of mRNA and 1 randomly selected lncRNA in maturing neurons.** Pearson’s correlation coefficient (*R_array_*) based on the microarray data, was computed between SLR and days 2, 4, 6, 8 after maturation. Pearson’s correlation coefficient (*R_qPCR_*) based on qPCR, was computed between fold change and days 2, 4, 6, 8, 14 after maturation. Expression is shown in fold change ± SD relative to Day 2. Expression of GAPDH was used as a control/housekeeping gene to normalize mRNA and lncRNA expression. Statistically significant differences were tested using the Student’s *t-test* (*p<0.05, **p<0.01). Mean C_T_ value ± SD for the no template control (NTC) is indicated.
(PDF)

Table S6 **Validation and quantification of miRNAs in maturing neurons.** Pearson’s correlation coefficient (*R_array_*) based on the microarray data, was computed between SLR and days 2, 4, 6, 8 after maturation. Pearson’s correlation coefficient (*R_qPCR_*) based on qPCR, was computed between fold change and days 2, 4, 6, 8, 14 after maturation. Expression is shown in fold change ± SD relative to Day 2. Expression of GAPDH was used as a control/housekeeping gene to normalize miRNA expression. Statistically significant differences were tested using the Student’s *t-test* (*p<0.05, **p<0.01). Mean C_T_ value ± SD for the no template control (NTC) is indicated.
(PDF)
