# Supplementary material for: Expression Profiling of RNA Transcripts during Neuronal Maturation and Ischemic Injury
Source: PLoS One. 2014 Jul 25;9(7):e103525. doi: 10.1371/journal.pone.0103525 (PMC4111601; doi:10.1371/journal.pone.0103525)
Supplement: Figure S1 — Maturation of (A) E14 and (B) E16 derived mouse primary cortical neuronal cultures. Cultures were immunostained for neuronal markers (MAP2 and NeuN), a neuronal progenitor marker (Sox2), microglial marker (CD11b), oligodendrocyte marker (O4) and astrocyte marker (GFAP). Short neurites at the early stages of development (Day 2) gave rise to an extensive network of neurites by day 8. E14 cultures displayed presence of neuronal progenitors whereas E16 cultures showed presence of astrocytes. Scale bar represents 50 µm. (PDF) [file pone.0103525.s001.pdf]

Figure S1A

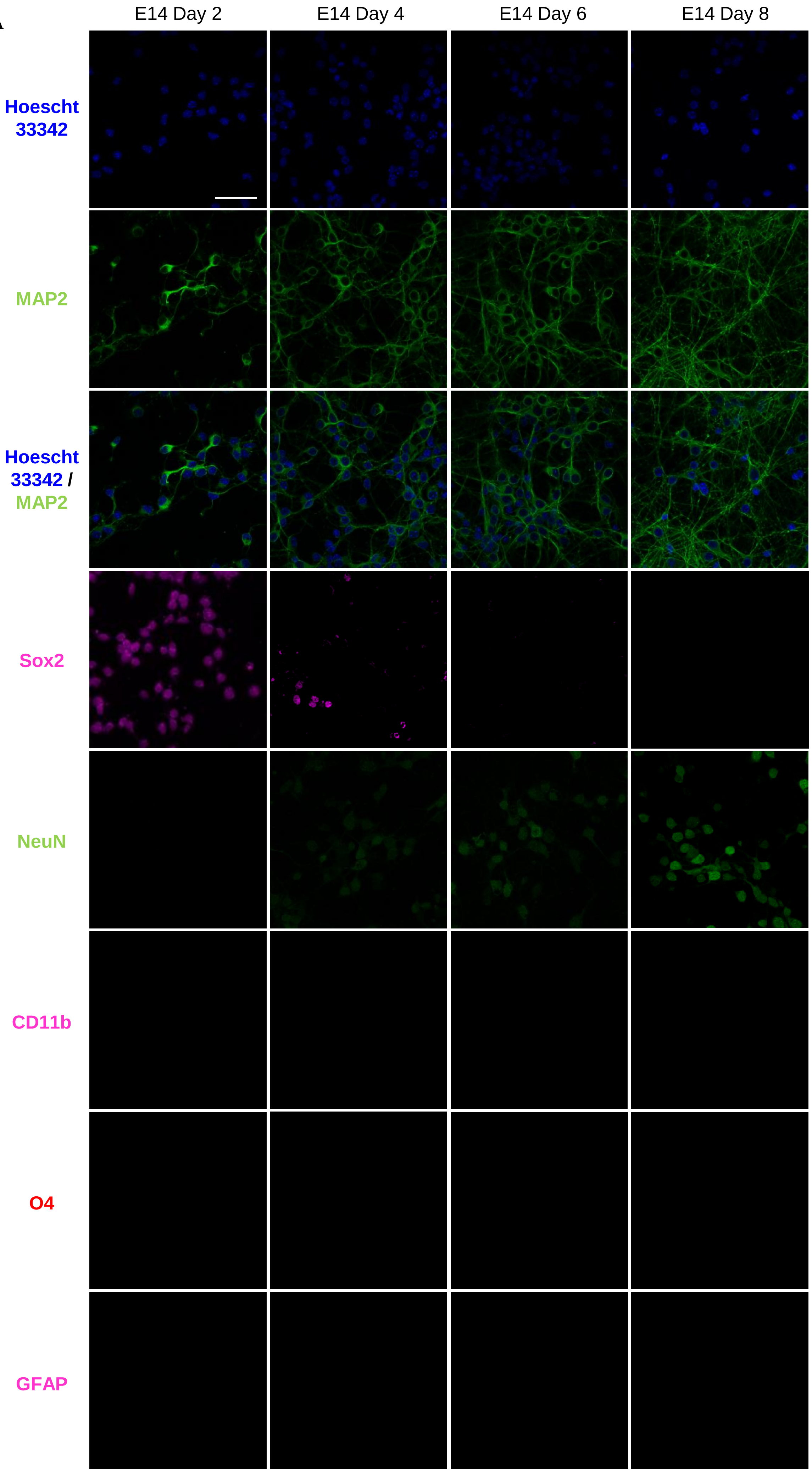
E14 Day 2
E14 Day 4
E14 Day 6
E14 Day 8
Hoescht 33342
MAP2
Hoescht 33342 / MAP2
Sox2
NeuN
CD11b
O4
GFAP

Figure S1B

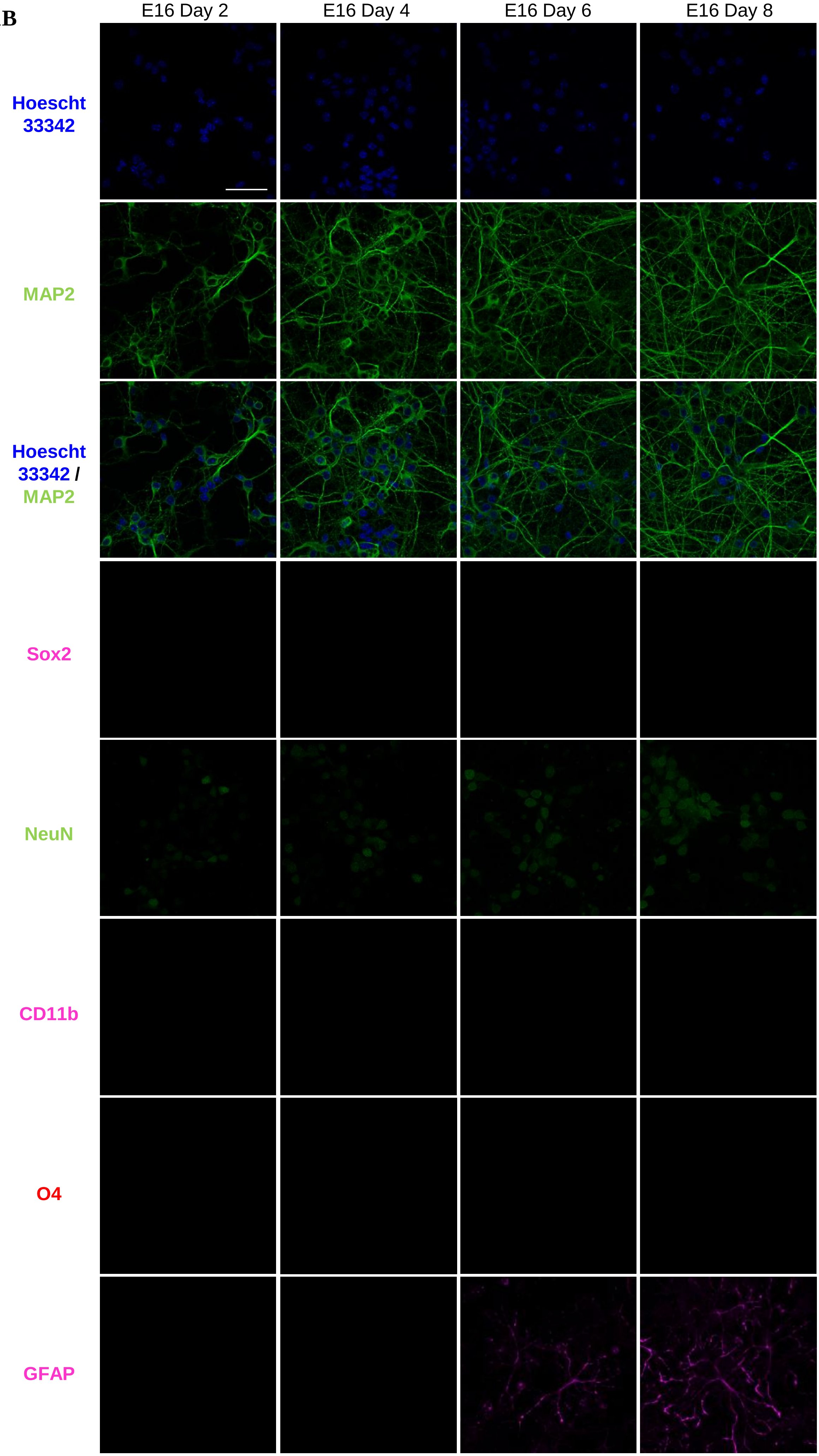
E16 Day 2
E16 Day 4
E16 Day 6
E16 Day 8
Hoescht 33342
MAP2
Hoescht 33342 / MAP2
Sox2
NeuN
CD11b
O4
GFAP

Figure S1. Maturation of (A) E14 and (B) E16 derived mouse primary cortical neuronal cultures. Cultures were immunostained for neuronal markers (MAP2 and NeuN), a neuronal progenitor marker (Sox2), microglial marker (CD11b), oligodendrocyte marker (O4) and astrocyte marker (GFAP). Short neurites at the early stages of development (Day 2) gave rise to an extensive network of neurites by day 8. E14 cultures displayed presence of neuronal progenitors whereas E16 cultures showed presence of astrocytes. Scale bar represents 50µm.
