# Supplementary material for: Expression Profiling of RNA Transcripts during Neuronal Maturation and Ischemic Injury
Source: PLoS One. 2014 Jul 25;9(7):e103525. doi: 10.1371/journal.pone.0103525 (PMC4111601; doi:10.1371/journal.pone.0103525)
Supplement: Figure S2 — Transcriptome of E15 derived primary cortical neuronal cultures (A) Differentially expressed genes in each cluster as identified in hierarchical clustering analysis of 6965 differentially expressed mRNAs (SLR>1) in maturing neurons ( Figure 2A ). Genes showing −6<SLR<−1.5 (cluster 1), 3.5<SLR<7.5 (cluster 2) on Day 6 or Day 8 are shown to represent the cluster. Green indicates downregulation and red, upregulation. (B) Differentially expressed lncRNAs in each cluster as identified in hierarchical clustering analysis of 7455 differentially expressed lncRNAs (SLR>1) in maturing neurons ( Figure 2B (i)). LncRNAs showing SLR = −8 (cluster 1), 2.4<SLR<3.4 (cluster 2), 1.3<SLR<2 (cluster 3), −4.5<SLR<−3 (Cluster 4) on day 6 or day 8 are shown to represent the cluster. Green indicates downregulation and red, upregulation. Genes associated with the lncRNAs in each cluster are stated in Table S3. (C) Differentially expressed miRNAs in each cluster as identified in hierarchical clustering analysis of 395 miRNAs after background subtraction of signal intensity less than 300 in maturing cortical neurons ( Figure 2C ). miRNAs showing −3<SLR<−0.3 (cluster 1), 1.2<SLR<2.2 (cluster 2), −0.1<SLR<−0.6 (Cluster 3) on day 6 or day 8 are shown to represent the cluster. Green indicates downregulation and red, upregulation. (PDF) [file pone.0103525.s002.pdf]

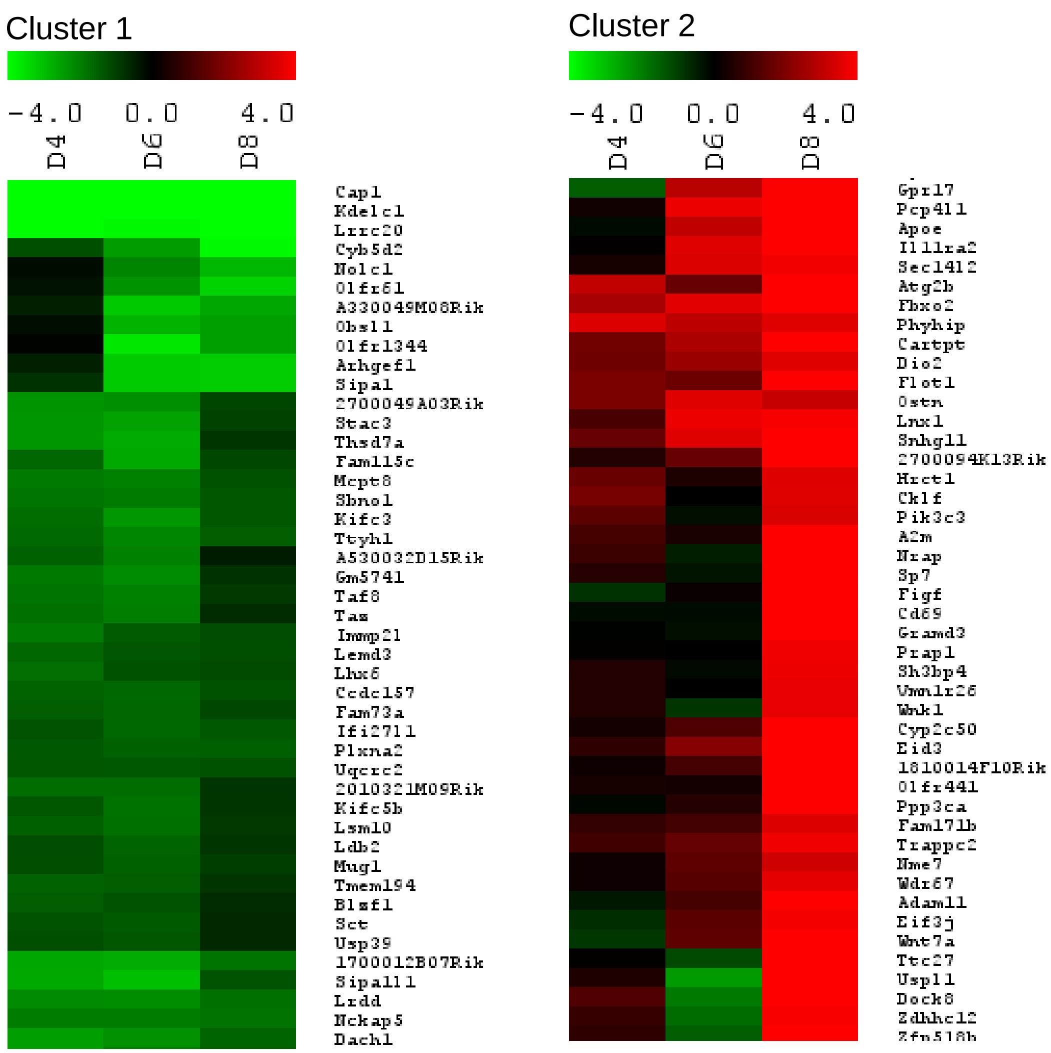

Figure S2A. Differentially expressed genes in each cluster as identified in hierarchical clustering analysis of 6965 differentially expressed mRNAs (SLR>1) in maturing neurons (Figure 2A). Genes showing -6<SLR<-1.5 (cluster 1), 3.5<SLR<7.5 (cluster 2) on Day 6 or Day 8 are shown to represent the cluster. Green indicates downregulation and red, upregulation.

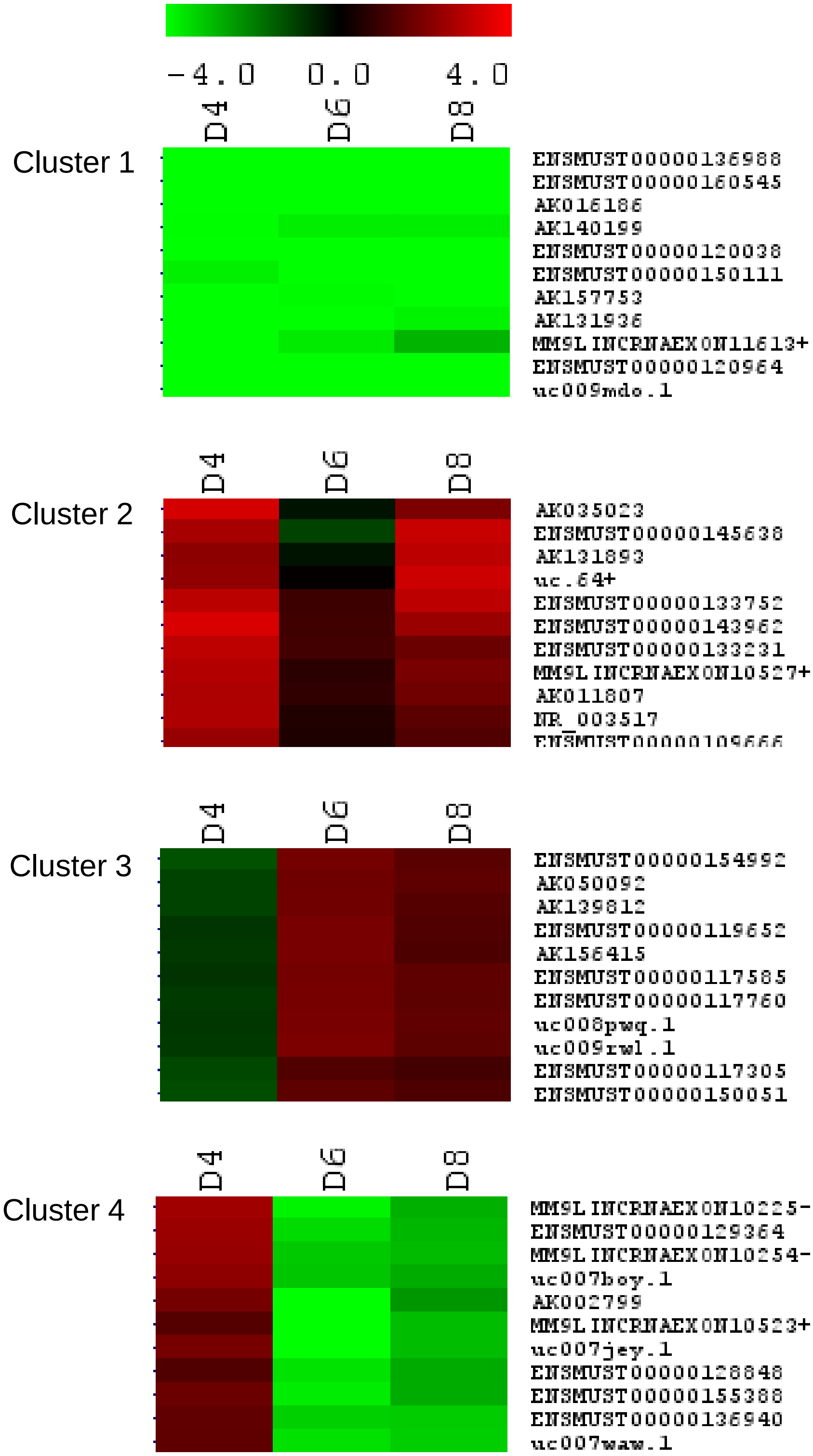

Figure S2B. Differentially expressed lncRNAs in each cluster as identified in hierarchical clustering analysis of 7455 differentially expressed lncRNAs (SLR>1) in maturing neurons (Figure 2B(i)). LncRNAs showing SLR=-8 (cluster 1), 2.4<SLR<3.4 (cluster 2), 1.3<SLR<2 (cluster 3), -4.5<SLR<-3 (Cluster 4) on day 6 or day 8 are shown to represent the cluster. Green indicates downregulation and red, upregulation. Genes associated with the lncRNAs in each cluster are stated in Table S3.

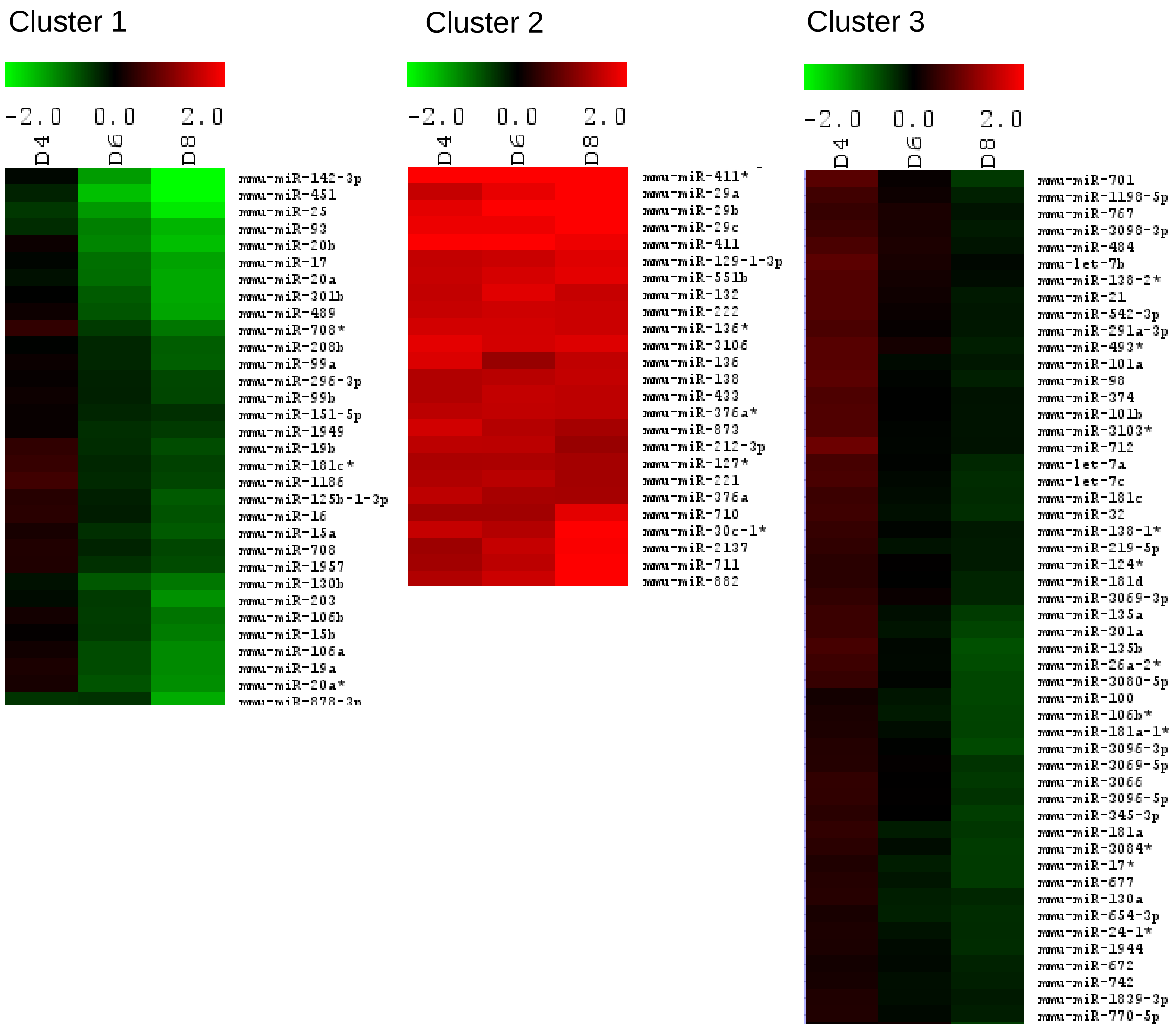

Figure S2C. Differentially expressed miRNAs in each cluster as identified in hierarchical clustering analysis of 395 miRNAs after background subtraction of intensity less than 300 in maturing cortical neurons (Figure 2C). miRNAs showing -3<SLR<-0.3 (cluster 1), 1.2<SLR<2.2 (cluster 2), -0.1<SLR<-0.6 (Cluster 3) on day 6 or day 8 are shown to represent the cluster. Green indicates downregulation and red, upregulation.
