# Supplementary material for: Expression Profiling of RNA Transcripts during Neuronal Maturation and Ischemic Injury
Source: PLoS One. 2014 Jul 25;9(7):e103525. doi: 10.1371/journal.pone.0103525 (PMC4111601; doi:10.1371/journal.pone.0103525)
Supplement: Figure S3 — Biological processes associated with differentially expressed (A) mRNAs, (B) lncRNAs associated with genes in sense/antisense/bidirectional orientation, (C) miRNAs. Green rectangle indicates fold enrichment for downregulated associated/predicted genes; red indicates fold enrichment of associated/predicted genes. (D) Biological processes which are common to the differentially expressed mRNAs, lncRNAs, and miRNAs. (PDF) [file pone.0103525.s003.pdf]

S3A

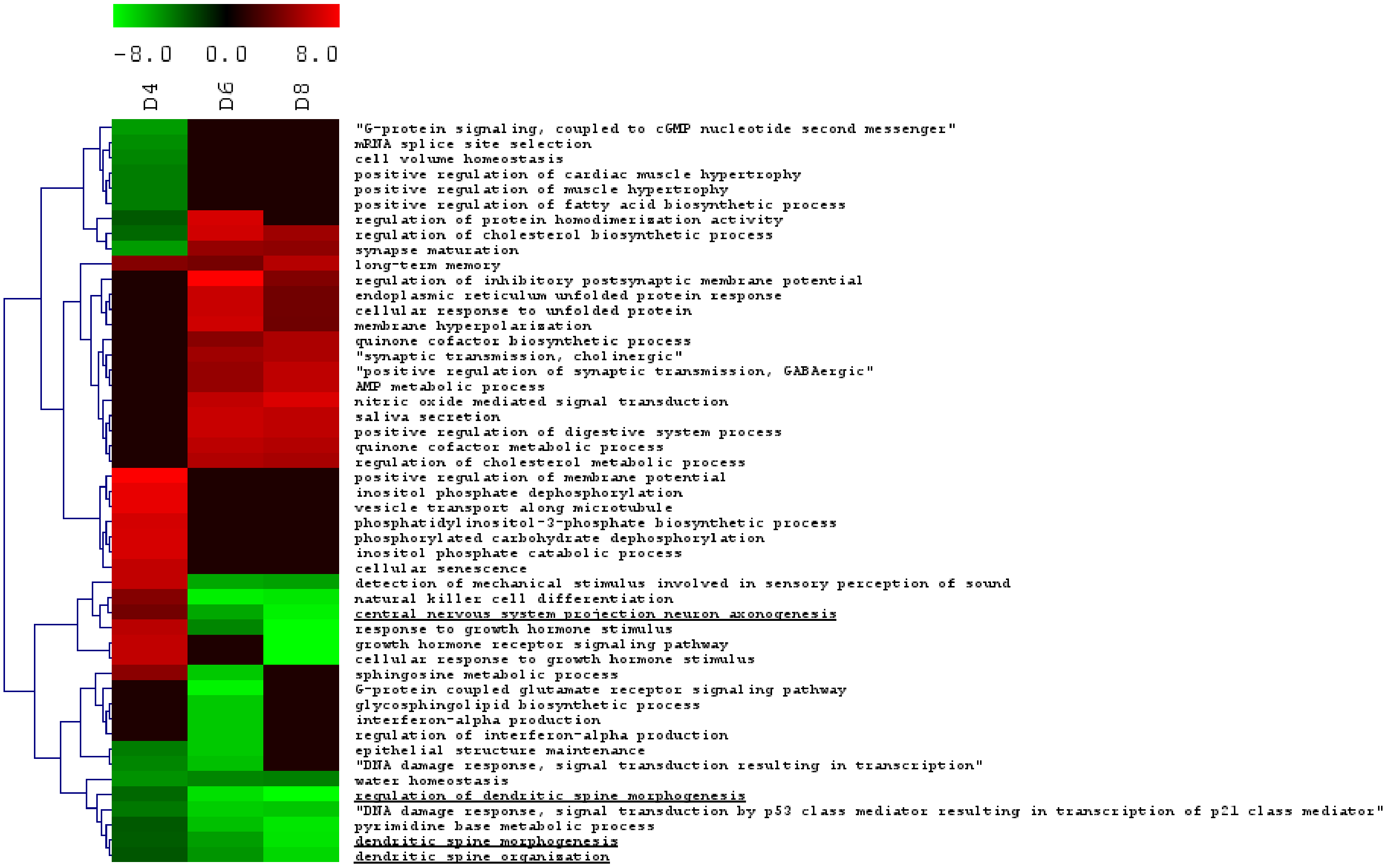

-8.0 0.0 8.0
D4
D6
D8
"G-protein signaling, coupled to cGMP nucleotide second messenger"
mRNA splice site selection
cell volume homeostasis
positive regulation of cardiac muscle hypertrophy
positive regulation of muscle hypertrophy
positive regulation of fatty acid biosynthetic process
regulation of protein homodimerization activity
regulation of cholesterol biosynthetic process
synapse maturation
long-term memory
regulation of inhibitory postsynaptic membrane potential
endoplasmic reticulum unfolded protein response
cellular response to unfolded protein
membrane hyperpolarization
quinone cofactor biosynthetic process
"synaptic transmission, cholinergic"
"positive regulation of synaptic transmission, GABAergic"
AMP metabolic process
nitric oxide mediated signal transduction
saliva secretion
positive regulation of digestive system process
quinone cofactor metabolic process
regulation of cholesterol metabolic process
positive regulation of membrane potential
inositol phosphate dephosphorylation
vesicle transport along microtubule
phosphatidylinositol-3-phosphate biosynthetic process
phosphorylated carbohydrate dephosphorylation
inositol phosphate catabolic process
cellular senescence
detection of mechanical stimulus involved in sensory perception of sound
natural killer cell differentiation
central nervous system projection neuron axonogenesis
response to growth hormone stimulus
growth hormone receptor signaling pathway
cellular response to growth hormone stimulus
sphingosine metabolic process
G-protein coupled glutamate receptor signaling pathway
glycosphingolipid biosynthetic process
interferon-alpha production
regulation of interferon-alpha production
epithelial structure maintenance
"DNA damage response, signal transduction resulting in transcription"
water homeostasis
regulation of dendritic spine morphogenesis
"DNA damage response, signal transduction by p53 class mediator resulting in transcription of p21 class mediator"
pyrimidine base metabolic process
dendritic spine morphogenesis
dendritic spine organisation

S3B

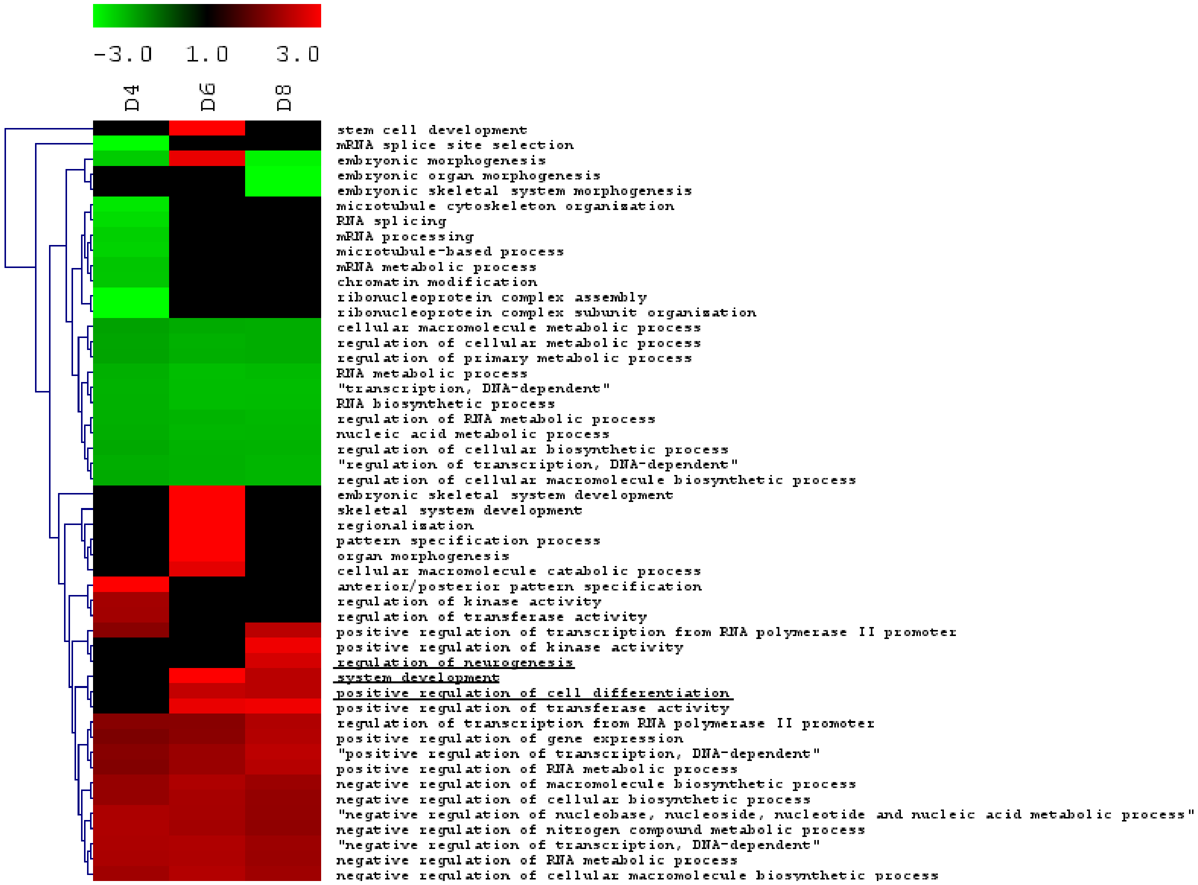

-3.0 1.0 3.0
D4
D6
D8
stem cell development
mRNA splice site selection
embryonic morphogenesis
embryonic organ morphogenesis
embryonic skeletal system morphogenesis
microtubule cytoskeleton organisation
RNA splicing
mRNA processing
microtubule-based process
mRNA metabolic process
chromatin modification
ribonucleoprotein complex assembly
ribonucleoprotein complex subunit organisation
cellular macromolecule metabolic process
regulation of cellular metabolic process
regulation of primary metabolic process
RNA metabolic process
"transcription, DNA-dependent"
RNA biosynthetic process
regulation of RNA metabolic process
nucleic acid metabolic process
regulation of cellular biosynthetic process
"regulation of transcription, DNA-dependent"
regulation of cellular macromolecule biosynthetic process
embryonic skeletal system development
skeletal system development
regionalization
pattern specification process
organ morphogenesis
cellular macromolecule catabolic process
anterior/posterior pattern specification
regulation of kinase activity
regulation of transferase activity
positive regulation of transcription from RNA polymerase II promoter
positive regulation of kinase activity
regulation of neurogenesis
system development
positive regulation of cell differentiation
positive regulation of transferase activity
regulation of transcription from RNA polymerase II promoter
positive regulation of gene expression
"positive regulation of transcription, DNA-dependent"
positive regulation of RNA metabolic process
negative regulation of macromolecule biosynthetic process
negative regulation of cellular biosynthetic process
"negative regulation of nucleobase, nucleoside, nucleotide and nucleic acid metabolic process"
negative regulation of nitrogen compound metabolic process
"negative regulation of transcription, DNA-dependent"
negative regulation of RNA metabolic process
negative regulation of cellular macromolecule biosynthetic process

S3C

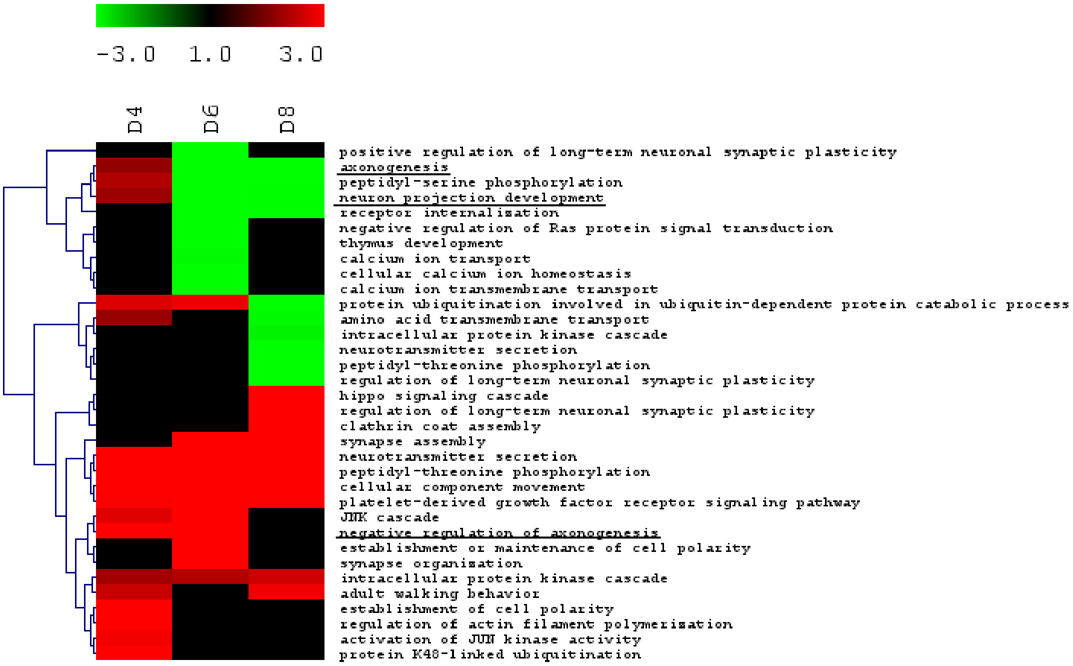

S3D

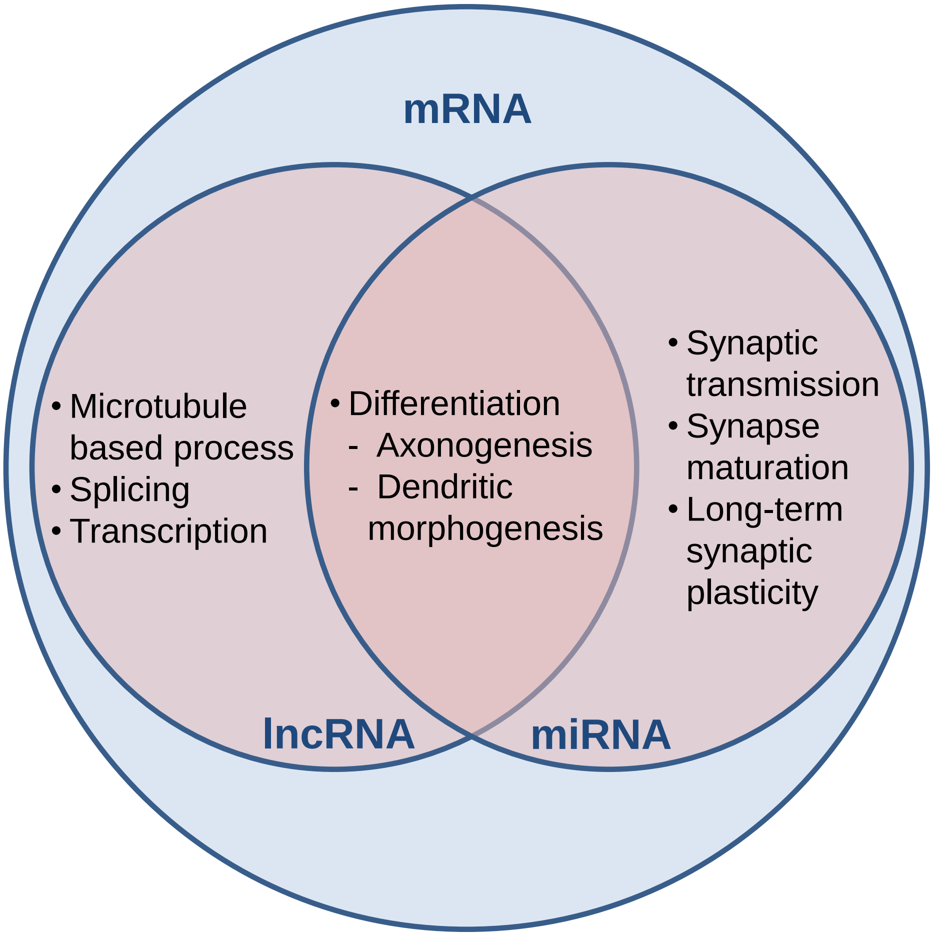

Figure S3. Biological processes associated with differentially expressed (A) mRNAs, (B) lncRNAs associated with genes in sense/antisense/bidirectional orientation, (C) miRNAs. Green rectangle indicates fold enrichment for downregulated associated/predicted genes; red indicates fold enrichment of associated/predicted genes. **(D)** Biological processes which are common to the differentially expressed mRNAs, lncRNAs, and miRNAs.
