# Supplementary material for: Expression Profiling of RNA Transcripts during Neuronal Maturation and Ischemic Injury
Source: PLoS One. 2014 Jul 25;9(7):e103525. doi: 10.1371/journal.pone.0103525 (PMC4111601; doi:10.1371/journal.pone.0103525)
Supplement: Table S1 — Specific primers for mRNAs and lncRNAs of 11 shortlisted genes. (PDF) [file pone.0103525.s004.pdf]

Table S1. Specific primers for mRNAs and lncRNAs of 11 shortlisted genes.

Gene		Accession No.	Forward primer (5' – 3')	Reverse primer (5' – 3')	Amplicon length
Axin2	mRNA	NM_015732.4	AGGTCCTGGCAACTCAGTAACAG	CGCGAACGGCTGCTTATT	61
	lncRNA	ENSMUST00000143435	GGTTCCACAGGCGTCATCTC	CCCGCAGCAGCTTTCTTC	55
Cntn1	mRNA	NM_001159648.1	CAAGTAGCTAGGGTGGGCTC	AAGACCTGTTCCCCACTCCT	139
	lncRNA	AK140484.1	CTGCAAAGGGAGTGGAAGCTA	GTAGGCAAGCAAATAGGCAGTGA	63
Igf1r	mRNA	NM_010513.2	GCGATTTAGAGAAACGAACATTCC	TGTCACGATGCCGGTTACC	82
	lncRNA	AK040698.1	AAGCACAGGACTTGGCAACAG	TTTCAAGCGAGGGCGTCTAG	60
Ikbkb	mRNA	NM_010546.2	TCTAAATGGCCTTTTCCTGCTAAT	TGACTCCCCAAAGTTAGATGCA	68
	lncRNA	uc009ldv.1	CATCCGGTGGCACAATCA	TTCCCGACAGGTCGTCTGA	59
Ncam1	mRNA	NM_010875.3	CGCCCCAGCTTGCCTACT	GAAGTTCCTGGCTTTTTCATAAACA	65
	lncRNA	AK156022	CTCTACAAGTTCAAGCGTTTGCA	GTGACTTTACGTCTGCCAAGTGA	65
Negr1	mRNA	NM_001039094	TGAAGCAGCGTGGGATACAAT	CCAGCGATTCCACAGACAAA	67
	lncRNA	uc008rva.1	CATCACCACCCTGCAGAAGA	GCTGCAAACCTCTCCGTTAATT	60
Nrxn1	mRNA	NM_020252	GTCAGCACTCAGGCATTGGA	CTTCTTGCGTGTAGCCCGTT	81
	lncRNA	uc008dwg.1	GGCTGATCTCCCACCTCATT	TCTGCTACTGGTTTAGTCCACA	109
Ntrk2	mRNA	NM_008745.3	TGTTGCCTATCCCAGGAAGTG	CTGCAGACATCCTCGGAGATTA	75
	lncRNA	AK021278.1	GAGATGTACTGCTGACTCCAAGCA	TCCTTGCACCCGCACTGT	62
Prkcb	mRNA	NM_008855.2	CAGAGATTGCCATCGGTCTGT	CCCCTCAGAATCCAGCATCA	92
	lncRNA	ENSMUST00000118119	GCAGAGGTTGGGTCCAAGTC	TGGGTGACCGGCAAATGT	61
Ralgds	mRNA	NM_009058.2	AGAGAAGCCCTCTCGTGAACTG	GGTGGTCAATGGCACTTTCAG	69
	lncRNA	uc008iyq.1	CAGGAAAAGAAGGTGGGCGAC	ATGCTCTTGTACATGTTGCC	77
Sh2b3	mRNA	NM_008507.3	CAGCTTGTGACGTCCGACTCT	GGACGGTGTTGGAGGAACCT	72
	lncRNA	AK007127.1	GAGAGACCTGCTCTGGAAAG	GGTTTCTCCCACTTCTGCGA	94

Specific accession numbers of the mRNAs and lncRNAs are indicated with forward and reverse primers to amplify the specific transcript. Length of the amplified PCR product is also indicated.
