# Supplementary material for: Expression Profiling of RNA Transcripts during Neuronal Maturation and Ischemic Injury
Source: PLoS One. 2014 Jul 25;9(7):e103525. doi: 10.1371/journal.pone.0103525 (PMC4111601; doi:10.1371/journal.pone.0103525)
Supplement: Table S2 — MIQE checklist for authors, reviewers and editors. Essential (E) and desirable information (D) has been made available in this table. Further details have been described in the main manuscript. (PDF) [file pone.0103525.s005.pdf]

Table S2. MIQE checklist for authors, reviewers and editors. Essential (E) and desirable information (D) has been made available in this table. Further details have been described in the main manuscript.

ITEM TO CHECK	IMPORTANCE	CHECKLIST
**EXPERIMENTAL DESIGN**		
Definition of experimental and control groups	E	Yes
Number within each group	E	Yes
Assay carried out by core lab or investigator's lab?	D	Yes
Acknowledgement of authors' contributions	D	Yes
**SAMPLE**		
Description	E	
Volume/mass of sample processed	D	Yes
Microdissection or macrodissection	E	No
Processing procedure	E	No
If frozen - how and how quickly?	E	No
If fixed - with what, how quickly?	E	No
Sample storage conditions and duration (especially for FFPE samples)	E	No
**NUCLEIC ACID EXTRACTION**		
Procedure and/or instrumentation	E	Yes
Name of kit and details of any modifications	E	Yes
Source of additional reagents used	D	Not applicable
Details of DNase or RNAse treatment	E	Yes
Contamination assessment (DNA or RNA)	E	Yes
Nucleic acid quantification	E	Yes
Instrument and method	E	Yes
Purity (A260/A280)	D	Yes
Yield	D	Yes
RNA integrity method/instrument	E	Yes
RIN/RQI or Cq of 3' and 5' transcripts	E	No
Electrophoresis traces	D	Yes
Inhibition testing (Cq dilutions, spike or other)	E	Yes
**REVERSE TRANSCRIPTION**		
Complete reaction conditions	E	Yes
Amount of RNA and reaction volume	E	Yes
Priming oligonucleotide (if using GSP) and concentration	E	Yes
Reverse transcriptase and concentration	E	Yes
Temperature and time	E	Yes
Manufacturer of reagents and catalogue numbers	D	No
Cqs with and without RT	D*	Yes
Storage conditions of cDNA	D	Not applicable
**qPCR TARGET INFORMATION**		
If multiplex, efficiency and LOD of each assay.	E	Not applicable
Sequence accession number	E	Yes
Location of amplicon	D	Yes
Amplicon length	E	Yes
In silico specificity screen (BLAST, etc)	E	Yes
Pseudogenes, retropseudogenes or other homologs?	D	No
Sequence alignment	D	No
Secondary structure analysis of amplicon	D	No
Location of each primer by exon or intron (if applicable)	E	Not applicable
What splice variants are targeted?	E	Not applicable

*: Assessing the absence of DNA using a no RT assay is essential when first extracting RNA. Once the sample has been validated as RDNA-free, inclusion of a no-RT control is desirable, but no longer essential.
**: Disclosure of the probe sequence is highly desirable and strongly encouraged. However, since not all commercial pre-designed assay vendors provide this information, it cannot be an essential requirement. Use of such assays is advised against.

Table S2. MIQE checklist for authors, reviewers and editors. Essential (E) and desirable information (D) has been made available in this table. Further details have been described in the main manuscript.

**qPCR OLIGONUCLEOTIDES**		
Primer sequences	E	Yes
RTPrimerDB Identification Number	D	No
Probe sequences	D**	No (Probe sequence not provided by manufactuer)
Location and identity of any modifications	E	Not applicable
Manufacturer of oligonucleotides	D	No
Purification method	D	No
**qPCR PROTOCOL**		
Complete reaction conditions	E	Yes
Reaction volume and amount of cDNA/DNA	E	Yes
Primer, (probe), Mg++ and dNTP concentrations	E	Yes
Polymerase identity and concentration	E	Yes
Buffer/kit identity and manufacturer	E	Yes
Exact chemical constitution of the buffer	D	No (Exact constitution of buffer not provided by manufacturer)
Additives (SYBR Green I, DMSO, etc.)	E	Yes
Manufacturer of plates/tubes and catalog number	D	No
Complete thermocycling parameters	E	Yes
Reaction setup (manual/robotic)	D	Yes (Manual)
Manufacturer of qPCR instrument	E	Yes
**qPCR VALIDATION**		
Evidence of optimisation (from gradients)	D	No
Specificity (gel, sequence, melt, or digest)	E	Yes
For SYBR Green I, Cq of the NTC	E	Yes
Standard curves with slope and y-intercept	E	Yes
PCR efficiency calculated from slope	E	Yes (106%)
Confidence interval for PCR efficiency or standard error	D	No
r2 of standard curve	E	Yes (0.99)
Linear dynamic range	E	Yes (1 to 10ng)
Cq variation at lower limit	E	Yes (1.9)
Confidence intervals throughout range	D	No
Evidence for limit of detection	E	Yes (at no template control level)
If multiplex, efficiency and LOD of each assay.	E	Not applicable
**DATA ANALYSIS**		
qPCR analysis program (source, version)	E	Yes
Cq method determination	E	Yes
Outlier identification and disposition	E	Yes
Results of NTCs	E	Yes
Justification of number and choice of reference genes	E	Yes
Description of normalisation method	E	Yes
Number and concordance of biological replicates	D	Yes
Number and stage (RT or qPCR) of technical replicates	E	Yes
Repeatability (intra-assay variation)	E	Yes
Reproducibility (inter-assay variation, %CV)	D	No
Power analysis	D	No
Statistical methods for result significance	E	Yes
Software (source, version)	E	Yes
Cq or raw data submission using RDML	D	No

*: Assessing the absence of DNA using a no RT assay is essential when first extracting RNA. Once the sample has been validated as RDNA-free, inclusion of a no-RT control is desirable, but no longer essential.

**: Disclosure of the probe sequence is highly desirable and strongly encouraged. However, since not all commercial pre-designed assay vendors provide this information, it cannot be an essential requirement. Use of such assays is advised against.
