# Supplementary material for: Expression Profiling of RNA Transcripts during Neuronal Maturation and Ischemic Injury
Source: PLoS One. 2014 Jul 25;9(7):e103525. doi: 10.1371/journal.pone.0103525 (PMC4111601; doi:10.1371/journal.pone.0103525)
Supplement: Table S3 — Associated gene names of lncRNAs for each cluster indicated in Figure S2B. (PDF) [file pone.0103525.s006.pdf]

Table S3. Associated gene names of lncRNAs for each cluster indicated in Figure S2B.

Cluster	LncRNA sequence name	Associated gene name
1	ENSMUST00000136988	Intergenic
	ENSMUST00000160545	radial spoke head protein 3 homolog A
	AK016186	promethin
	AK140199	Intergenic
	ENSMUST00000120038	filensin
	ENSMUST00000150111	Intergenic
	AK157753	Intergenic
	AK131936	Intergenic
	MM9LINCRNAEXON11613+	Intergenic
	ENSMUST00000120964	Intergenic
	uc009mdo.1	mitochondrial tRNA modification GTPase GTPBP3, plasmalemma vesicle-associated protein
2	AK035023	protein phosphatase 1 regulatory subunit 12B
	ENSMUST00000145638	hypothetical protein LOC233812
	AK131893	condensin-2 complex subunit H2 isoform b
	uc.64+	EH domain-binding protein 1
	ENSMUST00000133752	palladin
	ENSMUST00000143962	fibroblast growth factor receptor substrate 3
	ENSMUST00000133231	Intergenic
	MM9LINCRNAEXON10527+	Intergenic
	AK011807	tetratricopeptide repeat protein 23 isoform 1
	NR_003517	protein SFI1 homolog
	ENSMUST00000109666	Intergenic
3	ENSMUST00000154992	peroxisomal multifunctional enzyme type 2
	AK050092	Intergenic
	AK139812	Intergenic
	ENSMUST00000119652	Intergenic
	AK156415	Intergenic
	ENSMUST00000117585	Intergenic
	ENSMUST00000117760	Intergenic
	uc008pwq.1	Intergenic
	uc009rwl.1	Intergenic
	ENSMUST00000117305	cytosolic carboxypeptidase 6 isoform 1
4	MM9LINCRNAEXON10225-	Intergenic
	ENSMUST00000129364	zinc finger protein 335
	MM9LINCRNAEXON10254-	Intergenic
	uc007boy.1	striated muscle-specific
	AK002799	cip1-interacting zinc finger protein
	MM9LINCRNAEXON10523+	Intergenic
	uc007jey.1	folliculin
	ENSMUST00000128848	inactive ubiquitin carboxyl-terminal hydrolase
	ENSMUST00000155388	homeobox protein Hox-B3
	ENSMUST00000136940	hypothetical protein LOC232035
	uc007waw.1	WNT1-inducible-signaling pathway protein 1
