# Supplementary material for: Expression Profiling of RNA Transcripts during Neuronal Maturation and Ischemic Injury
Source: PLoS One. 2014 Jul 25;9(7):e103525. doi: 10.1371/journal.pone.0103525 (PMC4111601; doi:10.1371/journal.pone.0103525)
Supplement: Table S4 — List of 23 differentially expressed mRNAs in the top 3 pathways and the differentially expressed miRNAs that were predicted to target them. (PDF) [file pone.0103525.s007.pdf]

Table S4. List of 23 differentially expressed mRNAs in the top 3 pathways and the differentially expressed miRNAs that were predicted to target them.

Pathway	Gene	Differentially expressed miRNAs predicted to target gene
**Proliferation and differentiation related pathways**	*Abl1*	miR-101a, -101b, -128, -132, -15a, -15b, -16, -195, -203, -212, -30a, -30b, -30d, -30e, -378, -7a, -7b
	Axin2	miR-107, -15b, -15a, -16, -195, -221, -222, -290-5p, -374, -495
	E2f2	miR-106a, -106b, -17, -20a, -20b, -21, -221, -222, -301b, -302a, -335-5p, -721, -495, -93
	Fgfr1	miR-15b, -15a, -16, -195, -214, -22, -328, -342-3p, -376b, -339-5p, -495
	Igf1r	let-7a, let-7b, let-7d, let-7f, let-7g, let-7i, miR-30a, -30b, -30d, -30e, -376b, -488, -98
	Ikbkb	miR-130b, -15b, -15a, -16, -17, -190, -195, -20a, -20b, -214, -218, -29a, -29b, -29c, -301b, -33, -721, -93
	Itgb1	miR-214, -29a, -29b, -29c
	Kit	miR-107, -128, -137, -15a, -15b, -16, -185, -19a, -19b, -195, -218, -221, -222, -301b, -361, -410, -431, -542-3p, -721, -882
	Prkcb	miR-129-5p, -203, -326, -433, -495, -873
	Ptgs2	miR-101b, -101a, -107, -129-5p, -132, -15a, -15b, -16, -195, -212, -33, -342-3p, -338-3p, -374, -381, -410, -411, -433, -7a, -7b, -873
	Ralgds	miR-124, -185, -214, -302a, -374, -882, -9
**Cell adhesion molecules**	*Cdh4*	miR-124, -129-5p, -132, -148b, -149, -212, -33
	Cntn1	miR-106a, -106b, -124, -129-5p, -136, -146b-5p, -153, -17, -191, -20a, -20b, -203, -204, -218, -25, -300, -326, -33, -361, -363-5p, -374, -376a, -376b, -377, -381, -410, -411, -488, -495, -873, -93
	F11r	miR-125b-5p, -214, -290-5p, -338-3p, -342-3p, -377, -410
	Ncam1	miR-128, -204, -290-5p, -377, -411, -431, -542-3p
	Negr1	miR-101a, -101b, -124, -129-5p, -135b, -136, -203, -21, -329, -362-3p, -377, -382, -7a, -7b, -9
	Nrxn1	miR-128, -129-5p, -137, -142-3p, -190, -208b, -218, -290-5p, -329, -335-5p, -338-3p, -339-5p, -342-3p, -374, -377, -495
	Nrxn3	miR-129-5p, -185, -214, -218, -495, -882
**Neurotrophin signaling pathway**	*Camk2d*	miR-106a, -106b, -129-5p, -130b, -135b, -136, -17, -185, -191, -20a, -20b, -203, -214, -30a, -30b, -30d, -30e, -361, -377, -381, -382, -495, -873, -882, -93
	Ntrk2	miR-101b, -101a, -124, -138, -329, -33, -431, -873
	Rapgef1	miR-124, -138, -149, -203, -411, -488, -495, -542-3p, -873
	Sh2b3	let-7b, let-7d, let-7f, let-7g, let-7i, -218, -30a, -30b, -30d, -30e, -326, -542-3p, -98
	Sort1	miR-135b, -142-3p, -146b, -149, -15b, -190, -195, -324-5p, -361, -542-3p, -9
