# Supplementary material for: Expression Profiling of RNA Transcripts during Neuronal Maturation and Ischemic Injury
Source: PLoS One. 2014 Jul 25;9(7):e103525. doi: 10.1371/journal.pone.0103525 (PMC4111601; doi:10.1371/journal.pone.0103525)
Supplement: Table S5 — Validation and quantification of mRNA and 1 randomly selected lncRNA in maturing neurons. Pearson’s correlation coefficient (Rarray) based on the microarray data, was computed between SLR and days 2, 4, 6, 8 after maturation. Pearson’s correlation coefficient (RqPCR) based on qPCR, was computed between fold change and days 2, 4, 6, 8, 14 after maturation. Expression is shown in fold change ± SD relative to Day 2. Expression of GAPDH was used as a control/housekeeping gene to normalize mRNA and lncRNA expression. Statistically significant differences were tested using the Student’s t-test (*p<0.05, **p<0.01). Mean CT value ± SD for the no template control (NTC) is indicated. (PDF) [file pone.0103525.s008.pdf]

Table S5. Validation and quantification of mRNA and 1 randomly selected lncRNA in maturing neurons. Pearson's correlation coefficient (R_{array}) based on the microarray data, was computed between SLR and days 2, 4, 6, 8 after maturation. Pearson's correlation coefficient (R_{qPCR}) based on qPCR, was computed between fold change and days 2, 4, 6, 8, 14 after maturation. Expression is shown in fold change ± SD relative to Day 2. Expression of GAPDH was used as a control/housekeeping gene to normalize mRNA and lncRNA expression. Statistically significant differences were tested using the two sample *t-test* (**p<0.05*, ***p<0.01*). Mean C_T value ± SD for the no template control (NTC) is indicated.

**Gene**	**mRNA**	**Microarray**	**qPCR (Fold change ± SD)**					**qPCR (C_T ± SD)**
	**lncRNA**	R_{array}	**D4**	**D6**	**D8**	**D14**	R_{qPCR}	**NTC**
Proliferation and differentiation related pathway								
Axin2	NM_015732	-0.83	-1.38 ± 0.01*	-1.34 ± 0.01*	-1.16 ± 0.01	-2.63 ± 0.17*	-0.88	Undetermined
	ENSMUST00000143435	0.28	1.08 ± 0.06	1.70 ± 0.06*	3.28 ± 1.04*	3.04 ± 1.52*	0.86	39.3 ± 0.7
Igf1r	NM_010513	0.54	-1.75 ± 0.01**	-2.08 ± 0.02**	-1.68 ± 0.02**	-1.24 ± 0.02**	0.00	Undetermined
	AK040698	-0.97	-1.65 ± 0.01**	1.08 ± 0.02*	1.27 ± 0.13*	-4.77 ± 0.01**	-0.71	Undetermined
Ikbkb	NM_010546	0.87	-1.27 ± 0.01*	-1.44 ± 0.02*	-1.32 ± 0.02*	-2.34 ± 0.02**	-0.95	Undetermined
	uc009ldv.1	-0.04	-1.35 ± 0.15	-1.68 ± 0.02**	-1.96 ± 0.02**	-1.90 ± 0.24**	-0.81	35.6 ± 0.7
Prkcb	NM_008855	0.95	1.88 ± 0.07**	2.01 ± 0.01**	2.99 ± 0.02**	11.48 ± 0.20**	0.99	36.8 ± 0.1
	ENSMUST00000118119	0.94	1.12 ± 0.01*	1.00 ± 0.01	-1.01 ± 0.01	1.90 ± 0.74*	0.83	Undetermined
Ralgds	NM_009058	-0.64	-1.21 ± 0.05*	-1.22 ± 0.01**	-1.02 ± 0.02	-1.40 ± 0.01**	-0.68	37.0 ± 0.1
	uc008iyq.1	-0.73	-1.31 ± 0.01**	-1.43 ± 0.01**	-1.58 ± 0.01**	-1.73 ± 0.02**	-0.89	33.9 ± 0.2
Cell adhesion molecules								
Cntn1	NM_001159648	-0.78	1.39 ± 0.06*	1.76 ± 0.05**	2.27 ± 0.03**	2.69 ± 0.05**	0.93	34.9 ± 0.1
	AK140484	0.47	-1.45 ± 0.01*	1.10 ± 0.01	1.07 ± 0.02	-1.40 ± 0.07*	-0.34	Undetermined
Ncam1	NM_010875	0.89	-1.05 ± 0.06	1.29 ± 0.01**	1.35 ± 0.02**	1.44 ± 0.04**	0.85	Undetermined
	AK156022	-0.41	1.03 ± 0.03	1.01 ± 0.02	-1.07 ± 0.03*	-2.32 ± 0.01**	-0.90	37.1 ± 0.1
Negr1	NM_001039094	0.96	1.46 ± 0.03**	2.60 ± 0.01**	2.82 ± 0.02**	2.15 ± 0.05**	0.62	Undetermined
	uc008rva.1	-0.53	1.09 ± 0.02*	1.02 ± 0.02	-1.09 ± 0.01**	-1.16 ± 0.04**	-0.85	Undetermined
Nrxn1	NM_020252	0.21	1.17 ± 0.01**	1.03 ± 0.02	1.07 ± 0.01**	1.69 ± 0.01**	0.86	Undetermined
	uc008dwg.1	-0.48	-1.63 ± 0.01**	-1.34 ± 0.03*	-1.55 ± 0.02**	-1.44 ± 0.02*	-0.44	Undetermined
Neurotrophin signalling pathway								
Ntrk2	NM_008745	0.96	1.16 ± 0.08	2.06 ± 0.01**	3.82 ± 0.01**	4.90 ± 1.93**	0.93	Undetermined
	AK021278	-0.87	-1.50 ± 0.12*	-1.40 ± 0.07*	-1.46 ± 0.04*	-1.70 ± 0.12*	-0.78	37.1 ± 0.3
Sh2b3	NM_008507	0.84	-1.35 ± 0.02**	-1.14 ± 0.06	1.00 ± 0.06	1.44 ± 0.03**	0.79	37.0 ± 0.1
	AK007127	-0.53	-1.58 ± 0.03**	1.25 ± 0.01**	1.73 ± 0.08**	-6.64 ± 0.01**	-0.68	37.0 ± 0.2
