# Supplementary material for: Expression Profiling of RNA Transcripts during Neuronal Maturation and Ischemic Injury
Source: PLoS One. 2014 Jul 25;9(7):e103525. doi: 10.1371/journal.pone.0103525 (PMC4111601; doi:10.1371/journal.pone.0103525)
Supplement: Table S6 — Validation and quantification of miRNAs in maturing neurons. Pearson’s correlation coefficient (Rarray) based on the microarray data, was computed between SLR and days 2, 4, 6, 8 after maturation. Pearson’s correlation coefficient (RqPCR) based on qPCR, was computed between fold change and days 2, 4, 6, 8, 14 after maturation. Expression is shown in fold change ± SD relative to Day 2. Expression of GAPDH was used as a control/housekeeping gene to normalize miRNA expression. Statistically significant differences were tested using the Student’s t-test (*p<0.05, **p<0.01). Mean CT value ± SD for the no template control (NTC) is indicated. (PDF) [file pone.0103525.s009.pdf]

Table S6. Validation and quantification of miRNAs in maturing neurons. Pearson's correlation coefficient (R_{array}) based on the microarray data, was computed between SLR and days 2, 4, 6, 8 after maturation. Pearson's correlation coefficient (R_{qPCR}) based on qPCR, was computed between fold change and days 2, 4, 6, 8, 14 after maturation. Expression is shown in fold change ± SD relative to Day 2. Expression of GAPDH was used as a control/housekeeping gene to normalize miRNA expression. Statistically significant differences were tested using the two sample *t-test* (*$p<0.05$, **$p<0.01$). Mean C_T value ± SD for the no template control (NTC) is indicated.

	**Microarray**	**qPCR (Fold change ± SD)**					**qPCR (C_T ± SD)**
	R_{array}	**D4**	**D6**	**D8**	**D14**	R_{qPCR}	**NTC**
miR-124	-0.44	1.25 ± 0.01*	1.16 ± 0.09*	-1.12 ± 0.05	-1.70 ± 0.01**	-0.99	Undetermined
miR-129	-0.22	1.81 ± 0.04**	2.24 ± 0.03**	1.34 ± 0.02*	-4.80 ± 0.01**	-0.95	Undetermined
miR-218	-0.35	1.30 ± 0.03**	-1.07 ± 0.04*	1.31 ± 0.02**	1.23 ± 0.08**	0.15	Undetermined
miR-290-5p	0.15	-1.04 ± 0.19	1.16 ± 0.01**	1.36 ± 0.47	2.25 ± 1.09*	1.00	Undetermined
miR-326	0.44	1.43 ± 0.29	1.25 ± 0.02**	1.87 ± 0.01**	-1.26 ± 0.03*	-0.70	Undetermined
miR-203	-0.99	-1.67 ± 0.02**	-3.17 ± 0.03**	-3.16 ± 0.02**	-5.44 ± 0.01**	-0.93	Undetermined
miR-377	-0.99	12.51 ± 3.84*	12.00 ± 0.69**	5.13 ± 1.01*	-1.91 ± 0.01**	-0.98	Undetermined
